# The Next Generation Non-competitive Active Polyester Nanosystems for Transferrin Receptor-mediated Peroral Transport Utilizing Gambogic Acid as a Ligand

**DOI:** 10.1038/srep29501

**Published:** 2016-07-08

**Authors:** P. Saini, R. Ganugula, M. Arora, M. N. V. Ravi Kumar

**Affiliations:** 1 Department of Pharmaceutical Sciences, Texas A&M Health Science Center, Reynolds Medical Building, Mail Stop 1114, College Station 77843, Texas, USA.

## Abstract

The current methods for targeted drug delivery utilize ligands that must out-compete endogenous ligands in order to bind to the active site facilitating the transport. To address this limitation, we present a non-competitive active transport strategy to overcome intestinal barriers in the form of tunable nanosystems (NS) for transferrin receptor (TfR) utilizing gambogic acid (GA), a xanthanoid, as its ligand. The NS made using GA conjugated poly(lactide-co-glycolide) (PLGA) have shown non-competitive affinity to TfR evaluated in cell/cell-free systems. The fluorescent PLGA-GA NS exhibited significant intestinal transport and altered distribution profile compared to PLGA NS *in vivo*. The PLGA-GA NS loaded with cyclosporine A (CsA), a model peptide, upon peroral dosing to rodents led to maximum plasma concentration of CsA at 6 h as opposed to 24 h with PLGA-NS with at least 2-fold higher levels in brain at 72 h. The proposed approach offers new prospects for peroral drug delivery and beyond.

Receptor-mediated drug delivery using functional polymer nanosystems (NS) has been explored over the years to enhance the therapeutic index of drugs^1^,^2^,^3^,^4^,^5^. The recent use of receptor-mediated uptake in the gastrointestinal tract (GIT) presents an exciting opportunity for targeted and enhanced delivery of NS via vitamin B12^6,7^, folate-receptor^8^,^9^, neonatal fc receptor^10^ and transferrin-receptor (TfR)^11^,^12^,^13^. Due to its high expression, TfR is one of the prominent receptors explored for transport of NS across GIT^11^,^14^ and blood-brain barriers^15^,^16^ (BBB). TfR, responsible for the transport of iron bound transferrin across the GIT, is expressed in varying intensity across the GIT with the highest density in small intestine in villous cells of the epithelium^17^. Although TfRs are believed to be mainly concentrated on the basal and lateral sides of the epithelial cells in the small intestine, there are reports suggesting their presence on the apical side^17^ and concentration in clathrin-coated pits along the free cell margins^18^.

The ligands currently in use for receptor-mediated drug delivery must out-compete endogenous ligands in order to bind to the active site facilitating the transport^6^,^8^,^10^,^11^,^12^,^13^,^19^. Here, we present a novel non-competitive active transport strategy to overcome intestinal barriers by targeting TfR with tunable NS utilizing gambogic acid (GA), a xanthanoid, known for its affinity to TfR, independent of transferrin binding^20^,^21^. We have coupled GA to polylactide-co-glycolide (PLGA), the most widely used polymer in drug delivery^22^, via ethylenediamine (EDA) linker. The degree of ligand substitution on the NS surface is critical for receptor-mediated delivery, and can be optimized by controlling the mixing ratios of ligand-functionalized polymer to non-functional polymer^23^,^24^. This approach, utilizing small molecule non-competitive ligand for active drug delivery, enables the development of carrier systems that have no equivalent in the world of competitive ligands.

## Results and Discussion

### Synthesis and Characterization of GA-conjugated PLGA

**Figure 1:**
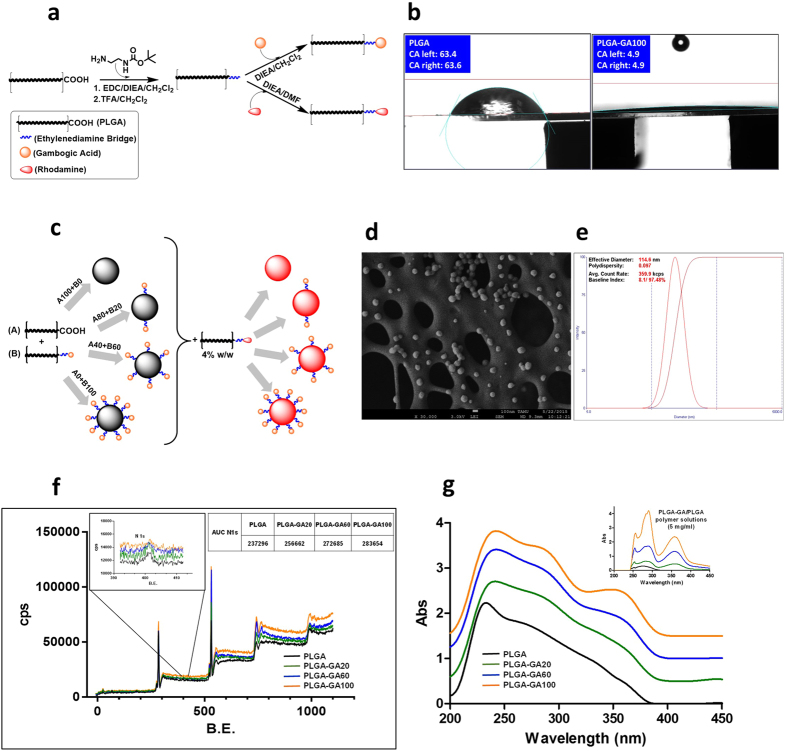
Synthesis and Characterization of GA-conjugated PLGA and PLGA-GA NS. (**a**) Schematic illustration of synthesis of PLGA-GA and PLGA-Rh. (**b)** Water droplet contact angle on PLGA and PLGA-GA100 (**c)** Engineered PLGA-GA/PLGA NS. (**d/e)** SEM and DLS analyses of the size of PLGA-GA100 NS. (**f)** Overlaid XPS survey spectra with an inset image of N 1 s scans of PLGA-GA/PLGA NS. (**g)** Overlaid UV/Vis absorption spectra of NS suspension with an inset image of the UV/Vis spectra of polymer solutions.

The carboxy-terminal end group of PLGA was activated with 1-ethyl-3-(dimethylaminopropyl)carbodiimide (EDC) and connected to *n-boc*-ethylenediamine via an amide bond, which was then de-protected to get PLGA-EDA with a free amine group. The amine functionality allowed for the formation of an amide-linkage to the carboxyl group of GA in the subsequent reaction (Fig. 1a). The covalent bonding of GA to PLGA was confirmed by NMR spectroscopy. The mild reactions employed in the conjugation process yield high amounts of products while preserving the structural integrity of the polymer backbone, as was evident from the GPC and MALDI-MS data, as well as unaltered thermal properties, observed in DSC (supplementary Fig. S1a–c).

The use of the biocompatible EDA linker^25^ to bind GA to PLGA served the dual purpose of activating PLGA end groups and imparting a high level of hydrophilicity^26^ to the ligand-terminus of the polymer chains. The hydrophilic end of the polymer chain facilitates the surface expression of ligands on NS during preparation in an aqueous environment. PLGA-GA itself exhibited higher hydrophilicity than PLGA in water contact angle analysis (Fig. 1b, supplementary Fig. S1d). In addition to PLGA-GA, rhodamine conjugated fluorescent PLGA-Rh was prepared by coupling rhodamine-NHS to PLGA-EDA.

### Preparation and Characterization of PLGA-GA NS

To evaluate the avidity of GA activated NS towards TfR, four variations of NS were engineered by blending PLGA-GA and PLGA in varying quantities: PLGA(100%); PLGA-GA(20%)- PLGA(80%); PLGA-GA(60%)- PLGA(40%) and PLGA-GA(100%) (Fig. 1c)(PLGA, PLGA-GA20, PLGA-GA60 and PLGA-GA100). PLGA-GA/PLGA blends in these concentrations yielded NS with 0, 4.04, 12.16 and 20.48 μg GA/mg PLGA respectively. The NS stabilized using minimal amounts of didodecyldimethylammonium bromide (DMAB) surfactant exhibited a spherical morphology with a diameter of ~110 nm (PDI ~0.1), which remained unchanged for all four variations of NS (Fig. 1d–e, supplementary Fig. S2a,b). Due to the interaction of the hydrophilic ligand-end of the polymer with the aqueous environment during the emulsification of organic phase carrying PLGA-GA, GA was expressed on the surface of the NS. An increasing trend of the amount of surface nitrogen in NS ranging from 0% to 100% PLGA-GA revealed by the x-ray photon spectra (XPS) indicated the surface expression of EDA, and hence the localization of GA on NS surface (Fig. 1f). It is ideal for the ligand to be expressed on the surface of the NS to provide access for the cell membrane receptors to bind to the ligands and internalize the NS. UV spectroscopy was also employed for surface characterization of PLGA-GA/PLGA NS following its established use to characterize adsorbed ligands on NS surface^27^,^28^ (Fig. 1g).

Incorporation of 4% (w/w) PLGA-Rh in the polymer mixture used to prepare NS yielded fluorescent NS (F-NS), fluorescing at excitation/emission (Ex/Em) wavelengths of 552/575 nm. The F-NS were employed in the cellular uptake, *ex vivo* intestinal tissue transport and *in vivo* distribution studies to track the NS.

In addition to non-fluorescent and fluorescent blank NS, cyclosporine A (CsA) loaded PLGA and PLGA-GA100 NS were prepared to evaluate CsA distribution kinetics in rodents. The NS prepared using the methods previously developed in our lab^29^ had the entrapment efficiency of 45% at 20% (w/w) loading or 90 μg CsA/mg polymer, irrespective of the conjugation status of the polymer. The NS displayed a slightly increased size of 157 nm due to the incorporation of CsA in the polymer matrix. The PLGA-GA and their NS were stable in gastric and intestinal conditions for the transit periods (supplementary Fig. S3a–c).

### ELISA Study to Evaluate Non-competitive Binding of PLGA-GA NS to TfR

**Figure 2:**
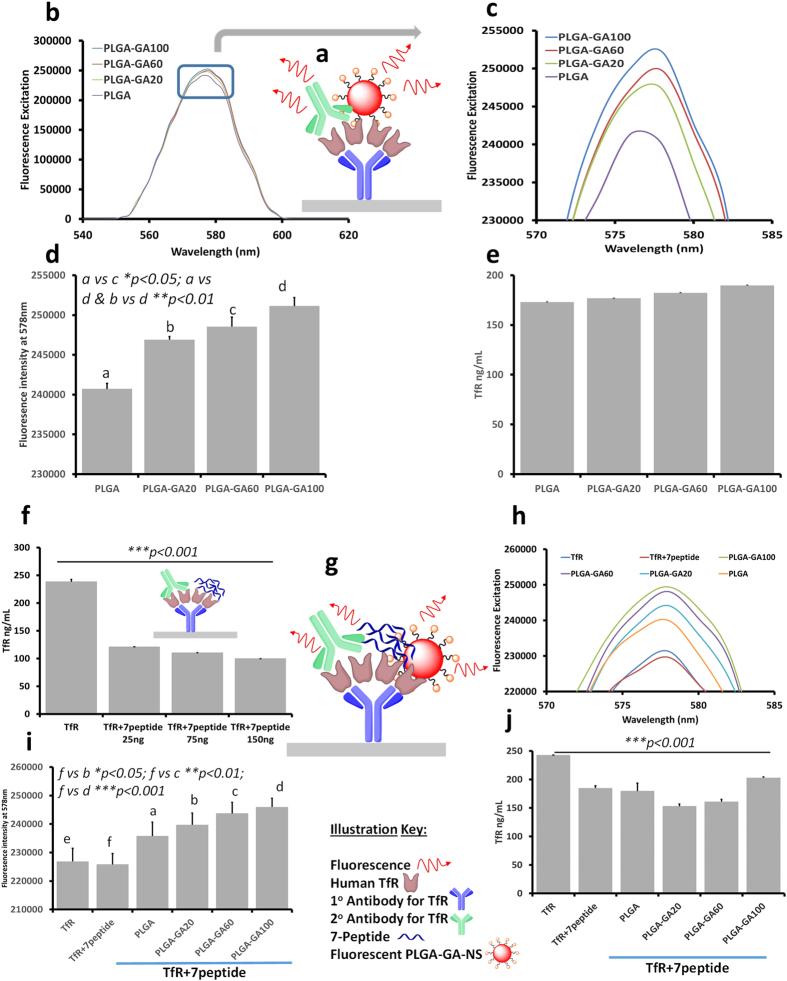
ELISA Study to Evaluate Non-competitive Binding of PLGA-GA NS to TfR. (**a**) Illustration of ELISA protocol used in the study. (**b**) Fluorescence excitation spectra of NS-receptor binding as a function of GA density. (**c**) Enlarged image of the boxed area in b. (**d**) Fluorescence intensity at 578 nm. (**e**) TfR levels after the binding of the 2° antibody. (**f**) TfR levels after binding of different amounts of 7-peptide to TfR. (**g**) Illustration of ELISA protocol used for competitive binding assay. (**h**) Fluorescence excitation spectra of NS-receptor binding as a function GA density in the presence 7-peptide. (**i**) Fluorescence intensity at 578 nm in the presence of 7-peptide. (**j**) TfR levels in competitive binding assay.

GA has been previously shown to have affinity for TfR, independent of transferrin binding 20. To evaluate the translation of the avidity of GA for TfR in PLGA-GA NS, a sandwich ELISA method was employed. The initial study involved the determination of the effect of surface GA density of NS in their binding affinity to TfR. Fluorescent PLGA, PLGA-GA20, PLGA-GA60 and PLGA-GA100 NS (F-NS) were introduced to 200 ng of TfR (Fig. 2a). After incubation and washing of the unbound NS, an increase in fluorescence intensity (FI) was seen with the increasing amount of PLGA-GA in the NS, and hence the surface density of GA. An increasing trend was observed in the height and area under the curves in wavelength scans for florescence excitation at 550-600nm as well as the FI values obtained at 578 nm, λ_max_ for PLGA-Rh. (Fig. 2b–d). The total FI of the NS suspension available in the wells without TfR was also measured in order to ensure that the variation in the FI with TfR was caused by the binding of the NS to the receptor (supplementary Fig. S4a-d). The initial FI, which was similar for all NS suspensions before the binding assay, was retained only by PLGA-GA100 at the end of the ELISA study, while the FI of others declined with the decrease in the amount of PLGA-GA in the NS. The similar FI for initial unwashed and final bound and washed PLGA-GA100 NS indicated that almost all the NS were bound to TfR. The colorimetric measurements for TfR levels depicted no significant reduction from the initial values signifying that GA does not compete with 2° antibody for TfR (Fig. 2e, supplementary Fig. S4e,f).

In the subsequent study, TfR was blocked using 7-peptide, a ligand with high affinity for TfR^11^ before treating with PLGA-GA/PLGA F-NS. Due to the lack of information available about the amount of 7-peptide required to completely block TfR, 250 ng of TfR was treated with 25, 75 and 150 ng of 7-peptide followed by estimating TfR levels using 2° TfR antibody. The colorimetric assay suggested no significant difference between the different amounts of 7-peptide used (Fig. 2f). Based on the results of the aforementioned assay, the highest tested 150 ng dose of 7-peptide was selected to sufficiently block TfR to evaluate the binding of PLGA-GA/PLGA NS to TfR in the presence of 2° antibody (Fig. 2g). Similar to the previous experiment devoid of a second ligand, an increase in FI was observed with the increasing surface GA density from PLGA to PLGA-GA100 (Fig. 2h,i). The increase in the binding capacity of PLGA-GA NS as a function of the surface density of GA was observed in spite of the decreased free TfR levels due to 7-peptide, confirmed by the colorimetric scans of the subsequently added 2° TfR antibody (Fig. 2j). Thus, it can be concluded that the ability of GA to bind non-competitively to TfR is preserved in the functionalized PLGA-GA NS. The DLS size distribution plots and SEM micrographs of PLGA or PLGA-GA100 NS at the dilutions used in the ELISA study are presented in supplementary Fig. S4g,h.

### Transport Studies of PLGA-GA NS with/without Blocking of TfR and Toxicity Evaluation in Cultured Cells

**Figure 3:**
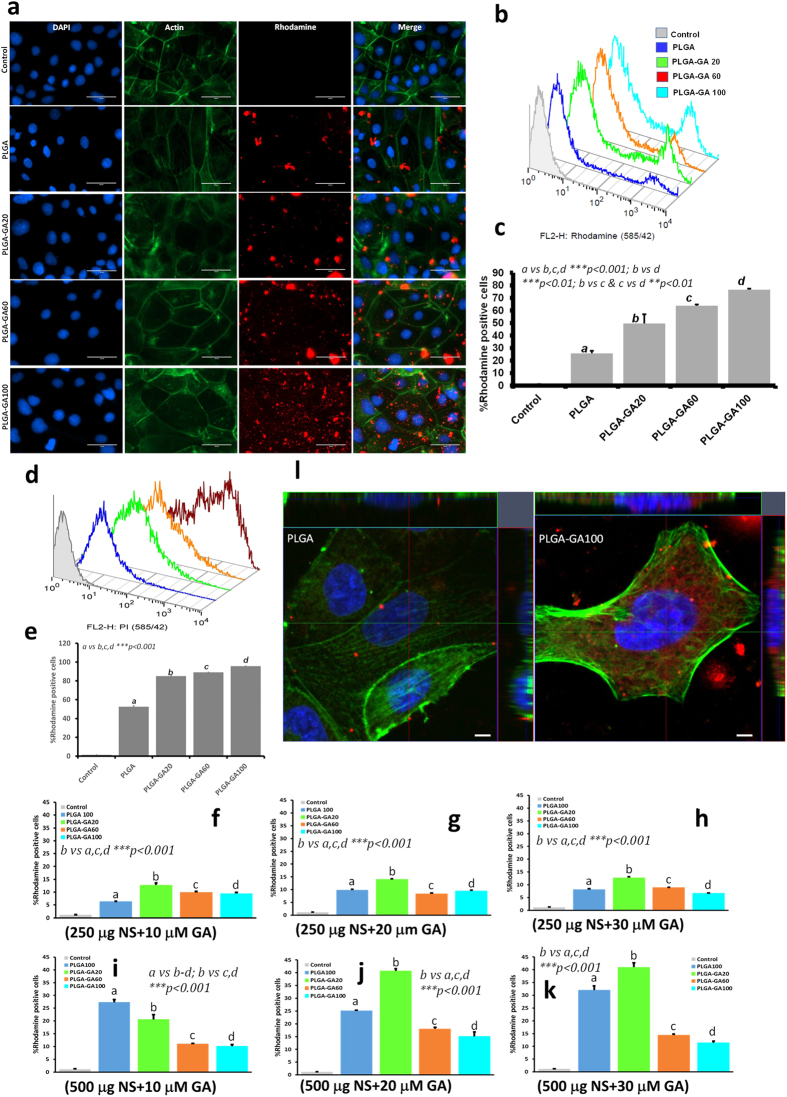
Transport Studies of PLGA-GA NS with/without Blocking of TfR and Toxicity Evaluation in Cultured Cells. (**a)** Cellular uptake of PLGA-GA/PLGA NS (treated with 500 μg/mL FL-NS) (red) with cytoskeleton and nuclei labeled with green and blue (DAPI) respectively and imaged using EVOS-FL microscope (60X, scale bar represents 50 μm). (**b/c**) FACS plots and quantification of Rh + cells. (**d/e**) Uptake of PLGA-GA NS across caco-2 cells on blocking TfR. (**f–h**) Histograms (FACS data) of rhodamine positive cells treated with 250 μg PLGA-GA/PLGA NS after pre-treatment with 10, 20 and 30 μM GA. (**i–k**) Histograms (FACS data) of rhodamine positive cells treated with 500 μg PLGA-GA/PLGA NS after pre-treatment with 10, 20 and 30 μM GA. (**l**) Confocal laser scanning microscopy images of caco-2 cells treated with PLGA and PLGA-GA NS (merged images, NS-red (rhodamine); nucleus-DAPI; actin (green); bar represents 5 μm. Images were analyzed using Zen 2012 software, where a median filter was applied followed by orthogonal sectioning to obtain sections of the XZ (green line, top margin) and YZ planes (red line, right margin) through the stack.

Caco-2 (human colon adenocarcinoma cell line) cells are extensively used as *in vitro* intestinal barrier model as they exhibit differentiated brush border on the apical surface with the expression of intestinal features like microvillus hydrolases, nutrient transporters and receptors including those for transferrin^30^,^31^. The cellular uptake of PLGA-GA/PLGA NS followed a pattern similar to their binding affinity to TfR reflected in the ELISA study. It was observed that suspending the NS directly in media or PBS caused excessive agglomeration (supplementary Fig. S5), thus limiting the uptake by cells. To avoid aggregation, NS (250 μg or 500 μg) were suspended in water (200 μl) and added to the culture wells followed by media after 5 min to allow the cells to bind and internalize the freely suspended NS followed by incubation for 1 h. Fluorescence microscopic investigation of the cell monolayers treated with PLGA-GA/PLGA F-NS revealed an increase in the amount of cellular uptake of the NS with the increase in the surface expression of GA (Fig. 3a).

This finding was further substantiated by the increase in FI for rhodamine in the monolayers treated with NS with increasing amount of PLGA-GA in the NS (Fig. 3b,c). A rightward shift in the cell population plots with respect to control was observed in Fluorescence activated cell sorting/scanning (FACS) corresponding to the increasing amount of rhodamine positive (Rh + ) cells (Fig. 3c). The shift in cell population increased with the increase in the amount of PLGA-GA present in the NS, concurrent with the FI and F-NS distribution observed in the microscopic evaluation. In addition, a separate new intense population of Rh + cells could be seen in the FACS plots indicative of some cells taking up more NS than others. This intense population of Rh + cells was more pronounced for the monolayers treated with 500 μg NS. In general, the cellular uptake of NS was higher when treated with larger amount of NS. The images and FACS analysis for the cells treated with 250 μg NS are presented in supplementary Fig. S6.

To study the non-competitive affinity of PLGA-GA/PLGA NS to TfR in the presence of another ligand, the cells were pre-treated with anti TfR 1^o^ antibody followed by treatment with 500 μg of PLGA-GA/PLGA or PLGA F-NS. TfR blocking did not compromise the cellular uptake of any of the NS variations, confirming the non-competitive binding of GA to TfR (Fig. 3d,e). In another experiment, the cells were pre-treated with 10, 20 and 30 μM GA to saturate the GA site on the receptor. In this case, a significant reduction in the uptake of PLGA-GA60 and PLGA-GA100 NS was observed but not for PLGA and PLGA-GA20 NS which rely on the passive uptake mechanisms. These findings substantiate our claim that the PLGA-GA NS employ TfR, non-competitively for their internalization into cells (Fig. 3f-k, supplementary Fig. S7). The PLGA/PLGA-GA NS localization was further confirmed by confocal images (Fig. 3l).

### Concentration dependent toxicity in caco-2 cells

**Figure 4:**
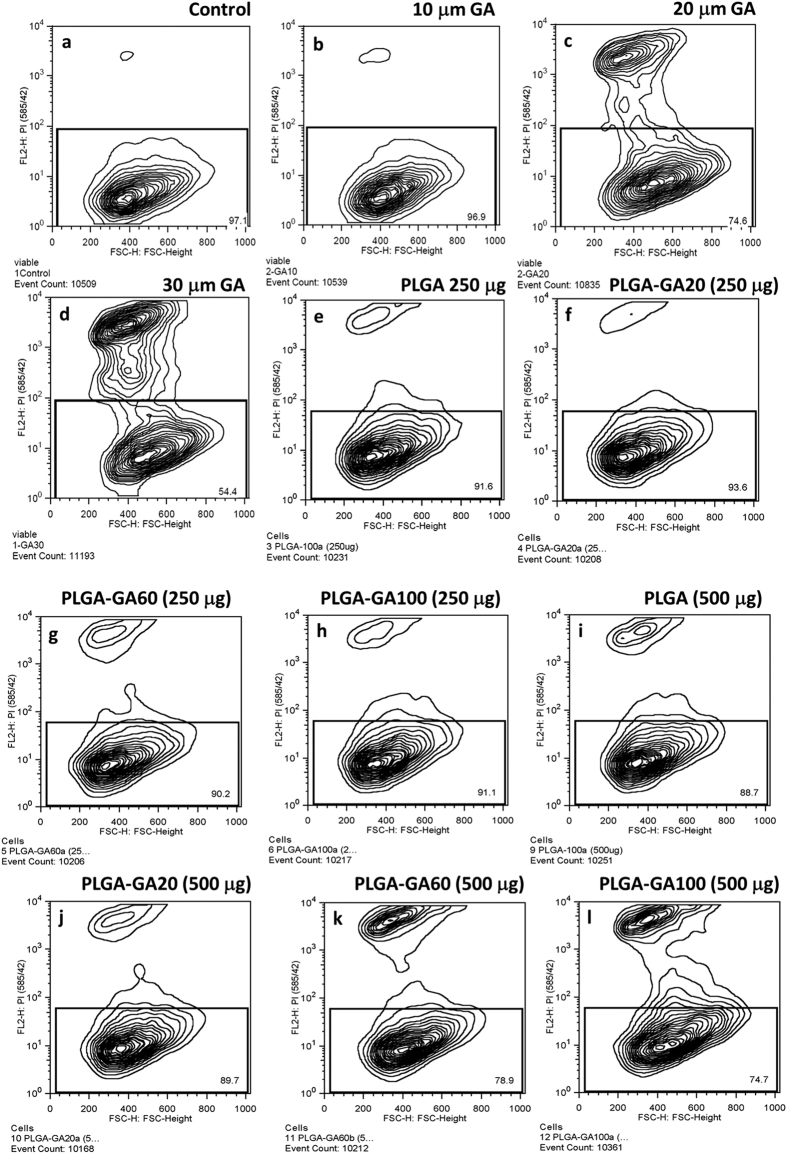
Concentration dependent toxicity in caco-2 cells. Contour plots of viable cells. (**a)** Control. (**b–d)** Increasing levels of cell death observed by increasing the amount of GA used to treat the cells with 10 μM treatment showing negligible cell death. (**e–h**) Cells treated with 250 μg PLGA-GA/PLGA NS with increasing amount of surface GA density showing negligible to very low cell death with no significant differences between groups. **(i–l)** Cells treated with 500 μg PLGA-GA/PLGA NS with increasing amount of surface GA density showing more cell death compared to 250 μg dose which may be due to the cumulative effect of excessive polymer and more GA available on the surface. The cell death was lower compared to the groups with 20 and 30 μM GA treated cells.

Since GA has been established as a potent anti-cancer agent^32^,^33^ with cytotoxic capabilities, it was imperative to evaluate the safety of PLGA-GA NS. While no cell death was observed in cells treated with 10 μM molecular GA, significant cell death of 25% and 46% was observed in those treated with 20 and 30 μM GA respectively (Fig. 4b–d). The cells treated with 250 μg of all PLGA-GA/PLGA NS and 500 μg of PLGA-100 and PLGA-GA20 did not show any significant cell death compared to control while 11% and 25% cell death was observed in case of cells treated with 500 μg PLGA-GA60 and PLGA-GA100 respectively (Fig. 4e–l). Cell death observed in the latter case could be attributed to the cumulative effect of high concentration of polymer and available GA. However, a moderate treatment of 250 μg NS appears to be safe for the cells. The amount of GA present in all the NS in 250 and 500 μg was much lower than the established safe dose of 60 mg/kg described in the literature^34^.

### *Ex vivo* Transport of PLGA-GA NS through Intestinal Barrier

**Figure 5:**
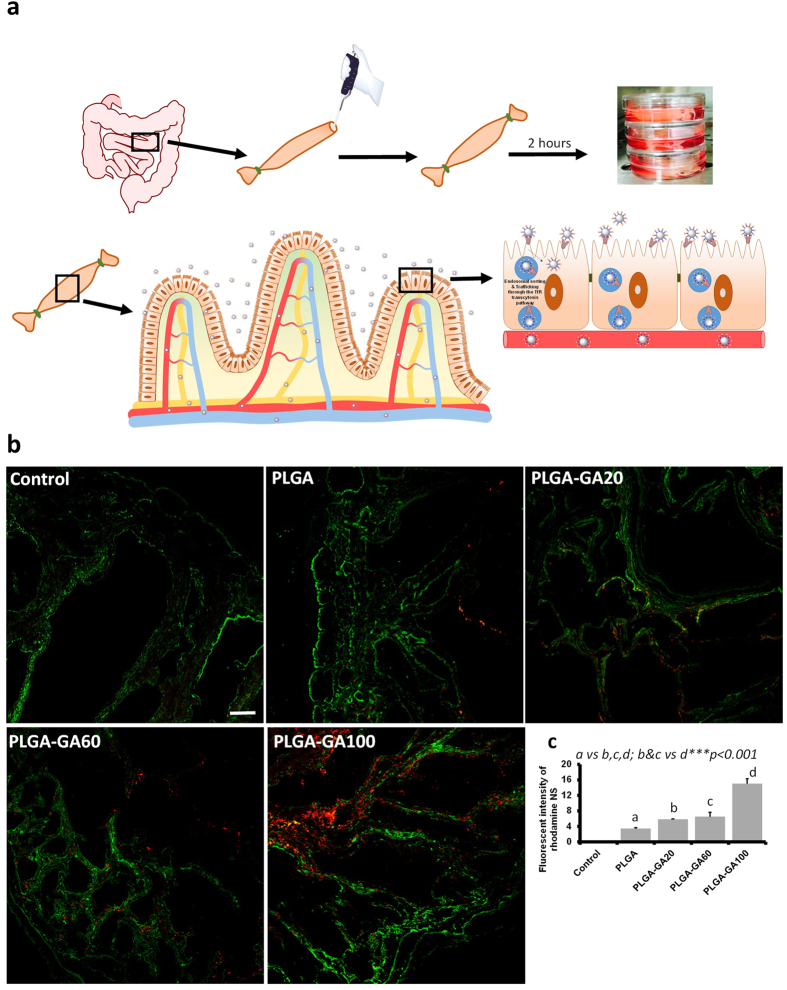
*Ex vivo* Transport of PLGA-GA NS through Intestinal Barrier. (**a**) Illustration of the experimental setup and mechanism of NS transport across intestinal tissue. (**b**) Representative fluorescence images of the *ex vivo* cryosections of jejunum obtained by CLSM (12×, scale bar represents 50 μm). TfR was stained using anti-TfR antibody (green). (**c**) Mean fluorescence intensity (MFI) quantified in the intestinal sections.

While caco-2 monolayers provide ample information about the receptor-mediated cellular uptake and safety of PLGA-GA NS, they do not mimic tissue-level transport and binding. To study the transport of the F-NS through the intestinal barriers, an *ex vivo* intestinal model^35^ (Fig. 5a) was employed by using a tubular section of jejunum of Sprague-Dawley (SD) rat, which is one of the intestinal areas with high density of TfR^17^. The sac like structure infused with NS (250 μg/ml) suspended in water was placed in media simulating physiological conditions for 2 h followed by washing and cryo-sectioning of the tissue for analysis (supplementary Fig. S8a). Concurrent with the observation in caco-2 monolayers, a large influx of NS into the intestinal tissue was observed for PLGA-GA NS with increasing amount surface GA density with highest uptake for PLGA-GA100 (Fig. 5b,c; supplementary Fig. S8b). The transport of the NS across the intestinal wall was confirmed by their appearance in the surrounding media via DLS and SEM analyses (supplementary Fig. S8c,d). These findings establish a clear role of active transport mediated by GA, since very low amount of F-NS without GA were absorbed by passive means.

### Kinetics of Fluorescent PLGA-GA NS

**Figure 6:**
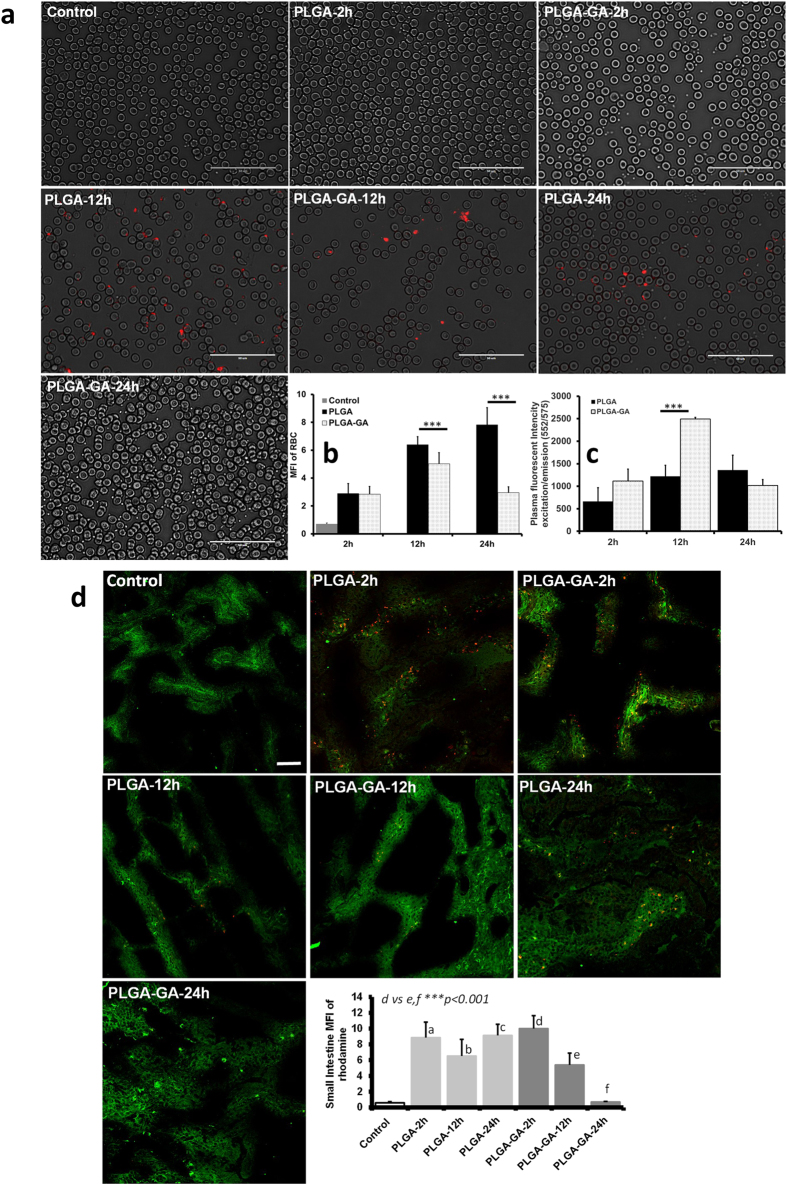
Kinetics of Fluorescent PLGA-GA NS. (**a**) Images of blood smears obtained using EVOS-FL microscope (60×, scale bar represents 50 μm). (**b**) MFI quantified in the blood smears. (**c**) Plasma fluorescence plotted as ex/em (552/575) profiles. Representative images and quantified MFI of rhodamine (red) in tissue sections (20×, scale bar represents 50 μm). Small intestine TfR is labelled green demonstrating the receptor presence on apical side.

The *in vivo* kinetics of PLGA-GA or PLGA F-NS were studied following a 50 mg/kg (~16 mg/rat) peroral dose and evaluation of red blood cells (RBC), plasma, small intestine, liver, spleen, kidneys and brain tissues for rats sacrificed at 2, 12 and 24 h. A gradual increase in RBC associated PLGA NS levels was observed over 24 h while PLGA-GA NS showed an initial increase until 12 h followed by a decline. The RBC associated PLGA-GA NS levels were lower than PLGA NS at all time points. Both NS displayed a similar concentration trend during 24 h in plasma as RBC, however the concentration of PLGA-GA NS was about 2-fold higher than PLGA NS at 12 h but slightly lower at 24 h (Fig. 6a–c, supplementary Fig. S9a–b). This finding suggests that PLGA-GA NS have a preferential residence in plasma over association with RBC during circulation, which can be attributed to the hydrophilic nature of PLGA-GA NS.

The presence of PLGA-GA NS in SI was highest at 2 h followed by a gradual decrease at 12 h with almost negligible amount present in the intestinal tissue at 24 h, indicative of complete transport across the intestinal barrier in the given timeframe (Fig. 6d, supplementary Fig. S9c). The fast absorption of PLGA-GA NS can be attributed to TfR mediated active transport. In comparison, a considerable amount of PLGA NS remained unabsorbed as indicated by their persistent levels observed in the tissue sections at all time points. A similar trend was observed in spleen where PLGA NS prevailed in high amounts until 24 h while PLGA-GA NS had a higher splenic concentration only at 2 h, but very low and decreasing concentrations at 12 and 24 h, indicating possible recirculation (supplementary Fig. S9d). In contrast, the concentration of PLGA-GA NS was highest in liver at 12 h with much lower concentrations at 2 and 24 h (supplementary Fig. S9e). The low levels of PLGA-GA NS in liver and spleen, the major organs of reticuloendothelial system (RES) responsible for clearance of foreign bodies including NS, reflect the recirculation of NS. Gustafson *et al*.^36^ cite examples in their review of nanomaterial phagocytosis, that a majority of NS reside within the RES organs for over 2 weeks after being cleared from circulation. While this effect was evident in the case of PLGA NS for the duration of the study, it was not observed for PLGA-GA NS. The recirculation of PLGA-GA NS was further affirmed by the appreciable levels of these NS in plasma at 24 h despite negligible concentration in small intestine, eliminating the possibility of transport of any residual unabsorbed NS from SI as in the case of PLGA NS. The hydrophilicity of PLGA-GA NS plays an integral part in their recirculation, as they can evade opsonization and hence accumulation in RES and phagocytosis by macrophages^37^,^38^,^39^,^40^. Renal clearance can also be ruled out for the NS considering their size which is larger than the renal filtration cut-off^41^ and their absence in glomerular areas in kidney sections (supplementary Fig. S9f).

TfR has been established as a resident receptor on the brain vasculature involved in the uptake and transport of transferrin and anti-TfR antibodies across the BBB^42^. TfR has been exploited as a potential target to deliver drugs^43^ and drug carrier systems^15^ across the BBB. In the present study, the PLGA-GA NS were able to penetrate the BBB as is evident by their accumulation in brain tissue in appreciable amount, with maximum concentration at 2 h followed by a statistically insignificant decline at 12 h and further decline at 24 h (supplementary Fig. S9g). In contrast, insignificant amount of PLGA NS was observed at 12 and 24 h. In comparison to PLGA NS, the concentration of PLGA-GA NS in brain accounted to about 7- and 4-folds at 2 and 12 h respectively.

### Cyclosporine A (CsA) Kinetics

**Figure 7:**
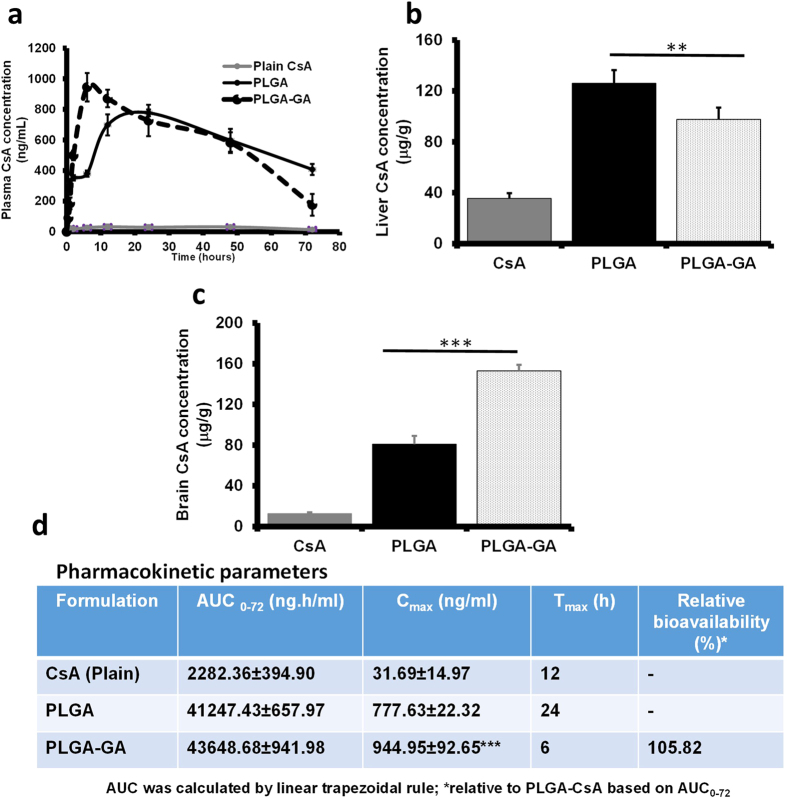
Cyclosporine A (CsA) Kinetics. (**a**) Plasma concentration profile of CsA over 72 h period (Grey: Plain CsA; Solid Black: PLGA NS CsA; Dotted Black: PLGA-GA NS CsA). (**b**) Liver distribution of CsA at the end of the study (72 h). (**c**) Brain distribution of CsA at the end of the study (72 h). (**d**) Pharmacokinetics parameters.

A drug kinetics study for encapsulated CsA was performed in SD rats following a single peroral dose of 15 mg/kg plain or encapsulated CsA in PLGA and PLGA-GA NS. Plasma concentration of CsA between 0–72 h revealed that a concentration maximum (C_max_) was reached at 12 h (maximum time taken, T_max_) for plain CsA and 24 h for PLGA NS, but much sooner at 6 h for PLGA-GA NS (Fig. 7a,d). This finding resonates with the results of the plasma concentration of F-NS with maximum FI at 24 and 12 h for PLGA and PLGA-GA NS respectively. The plasma concentration profiles, area under the curve (AUC_0–72_) and C_max_ for CsA were the highest for PLGA-GA NS indicative of a larger amount of these NS being transported across the intestinal barrier compared to PLGA NS and plain CsA. The significant decrease in T_max_ for PLGA-GA NS offers renewed hope for the PLGA delivery systems which otherwise are not suitable for drugs needing immediate release profiles or those with a narrow therapeutic index (e.g., CsA) where C_max_ can be detrimental for efficacy while maintaining the AUC. The concentration of CsA in liver at the termination of study (72 h) was lower for PLGA-GA NS than PLGA NS but higher than plain CsA (Fig. 7b). On the other hand, brain had a 2-fold higher concentration of CsA in PLGA-GA NS group than PLGA NS, following the trend observed *in vivo* F-NS distribution study (Fig. 7c). PLGA and PLGA-GA NS show an opposing trend of CsA concentration in brain compared to plasma where CsA concentration is higher for PLGA NS than PLGA-GA NS. This finding is indicative of enhanced absorption of PLGA-GA NS in tissues without being restricted to RES or plasma. Thus, it can be concluded that the PLGA-GA NS with encapsulated drug can effectively deliver the drug payload across the intestinal barrier and to target organs with high TfR density, like brain.

## Conclusions

We have described the use of GA, an established anti-tumor agent with a high affinity for TfR, as a non-competitive targeting moiety for peroral delivery of PLGA based NS. PLGA-GA NS are safe with the amount of GA used as the targeting agent and are internalized by cells expressing TfR. Blank and drug loaded PLGA-GA NS have the ability to cross the intestinal barrier to reach and stay in circulation for a long time, in part due to recirculation from organs, owing to their high hydrophilicity. Due to active transport of these NS, they can decrease the time taken (T_max_) to achieve concentration maximum (C_max_) for orally delivered encapsulated drugs compared to untargeted PLGA NS. In addition, PLGA-GA NS have the ability to penetrate the BBB in appreciable quantities. Thus, PLGA-GA NS offer a novel platform for peroral delivery of drugs utilizing a small molecule, non-competitive ligand for active drug delivery, enabling the development of carrier systems that have no equivalent in the world of competitive ligands.

## Methods

### Materials

PLGA 50:50 (Resomer 503H, viscosity 0.32–0.44 dl/g and MW 24,000–38,000) was purchased from Evonik (Darmstadt, Germany), n-boc-ethylenediamine from Combi-Blocks (San Diego, USA) and gambogic acid from Broadpharm (San Diego, USA). All other reagents, unless otherwise mentioned, were of analytical grade and were purchased from Fisher Scientific (USA).

Transferrin Receptor (TfR) ELISA assay kit was purchased from Eagle Biosciences (Nashua, USA). For cell culture experiments, caco-2 cells and Dulbecco’s Modified Eagle Medium (DMEM/F-12) were purchased from ATCC (Manassas, USA), human/mouse specific TfR primary antibody from Abcam (Cambridge, USA), ActinGreen and NucBlue molecular probes from Life technologies (Carlsbad, USA). All other reagents were cell culture grade and were purchased from Fisher Scientific (USA). ProLong Gold antifade mountant with DAPI was purchased from Life Technologies (Carlsbad, USA). CsA ELISA kit specific for rat was purchased from MyBioSource [MBS263904] (San Diego, USA).

### Synthesis of GA-conjugated PLGA

PLGA (500 mg; 0.0161 mmol) was stirred with 1-Ethyl-3-(dimethylaminopropyl) carbodiimide (EDC) (15.43 mg; 0.0805 mmol) in 5 ml CH_2_Cl_2_ for 30 min. Subsequently, n-boc-ethyelenediamine (12.90 mg; 12.75 μl; 0.0805 mmol) and N,N-Diisopropylethylamine (DIEA) (10.40 mg; 4.02 μl; 0.0805 mmol) were added and the reaction was continued under inert conditions for 18 h at room temperature. The reaction mixture was precipitated in 100 ml cold diethyl ether to get a white polymer. The polymer was further purified by re-dissolving it in 5 ml methylene chloride (CH_2_Cl_2_) and precipitating in cold diethyl ether. The purified polymer was dried under vacuum to get 483 mg of dry powder. The dried polymer was reconstituted in 3.3 ml 10:1 CH_2_Cl_2_:TFA solution and stirred under inert conditions for 1 h. The solvent was evaporated on a rotary evaporator to get a clear viscous residue which was dissolved in 3 ml CH_2_Cl_2_ and precipitated in cold ether. The polymer thus obtained was dried under vacuum to get 431 mg (86% theoretical yield) of dry PLGA-EDA. ^1^H NMR (500 MHz, CDCl_3_) δ ppm 1.54–1.62 (m, 3H), 2.80 (s, 2H), 3.78 (s, 2H), 4.69–4.90 (m, 2H), 5.15–5.31 (m, 1H).

To conjugate GA to PLGA-EDA, GA (25.84 mg; 0.0411 mmol) and EDC (9.46 mg; 0.043 mmol) were first dissolved in CH_2_Cl_2_ (1 ml) and stirred under inert conditions for 30 minutes. To the bright orange solution thus obtained, a solution of PLGA-EDA (424 mg; 0.0137 mmol) and DIEA (6.37 mg; 8.6 μl; 0.0493 mmol) in CH_2_Cl_2_ (2 ml) was added. The reaction mixture was further stirred for 18 h and then precipitated in 50 ml cold diethyl ether. The polymer was purified by dissolving in 3 ml CH_2_Cl_2_ and precipitating in cold diethyl ether. Finally, the polymer was washed with distilled water and dried under vacuum for 2 h to get 376 mg of yellow PLGA-GA (74% theoretical yield). ^1^H NMR (500 MHz, CDCl_3_) δ ppm 1.43–1.81 (m, 32H), 2.05 (m, 3H), 2.35 (m, 4H), 3.45–3.53 (m, 4H), 4.58–4.98 (m, 2H), 5.46–5.48 (m, 2H), 5.46–5.48 (m, 2H), 5.13–5.31 (m, 1H), 6.70 (m, 1H), 6.68 (m, 1H), 7.56–7.59 (m, 2H).

For the synthesis of PLGA-Rh, NHS-Rhodamine (1.7 mg; 0.0032 mmol), PLGA-EDA (50 mg; 0.0016 mmol) and DIEA (0.414 mg, 0.56 μl; 0.0032 mmol) were dissolved in DMF (1 ml) and stirred in an amber vessel under inert conditions for 12 h. The reaction mixture was the poured into 15 ml distilled water to precipitate the polymer. The polymer was further washed with distilled water twice to remove residual unreacted NHS-Rhodamine and dried under vacuum for 2 h to get 39 mg (76% theoretical yield) of pink colored PLGA-Rh.

### Characterization of GA-conjugated PLGA

All conjugated polymers and their intermediates were characterized for their chemical structure on a 500 MHz NMR. The molecular weights of the parent PLGA and conjugated PLGA-GA were evaluated using Agilent 1220 Infinity HPLC connected to Agilent 1260 Infinity refractive index detector (RID) to serve as a Gel Permeation Chromatography (GPC) system. The column used for the GPC analysis was Agilent PLgel 5μm MIXED-D column. The column was calibrated against polystyrene standards, and chloroform was used as a dissolving medium and eluent for both calibration standards and experimental samples.

Addition of GA to PLGA was also assessed using VWR 6300PC Double beam spectrophotometer. In addition to pure polymers, PLGA-GA/PLGA solution blends with 20% and 60% of PLGA-GA in the total weight of the mixture were analyzed. The pure polymer and their mixtures were dissolved in ethyl acetate and their absorbance spectra were scanned in the UV and visible light regions in a quartz cuvette.

Thermal analysis of PLGA and PLGA-GA was performed on TA Q50 differential scanning calorimetry (DSC) in the heat/cool/heat mode, where the polymer samples were heated at 10 °C/min from 0–200 °C and cooled at 5 °C/min in a covered aluminum pan under nitrogen environment.

In order to study the hydrophilicity of the aforementioned polymers and polymer mixtures, samples were prepared by coating glass coverslips with the polymers by solvent casting. 100 μl of each polymer solution in ethyl acetate at a concentration of 50 mg/ml was evenly spread on the coverslip and allowed to air dry followed by drying under vacuum to evaporate residual solvent. The contact angle of water droplets on the polymer surface was measured using Dataphysics OCA15EC contact angle measuring system.

### Preparation of PLGA-GA NS

Four 50 mg batches (n = 3) were prepared by mixing varying amounts of PLGA-GA and PLGA in the ratios that included PLGA (100%), PLGA (80%)-PLGA-GA (20%), PLGA (40%)-PLGA-GA (60%) and PLGA-GA (100%). To prepare the fluorescent NS, 4% (w/w) PLGA-Rh was added to the polymer mixtures.

The organic phase was prepared by dissolving 50 mg polymer in 2.5 ml ethyl acetate by stirring at 1000 rpm for 30 min. A 1% solution of N-dodecyl-N,N-dimethylammonium bromide (DMAB) in 5 ml distilled water was prepared by stirring at 1000 rpm for 20 min to serve as the aqueous phase. The organic phase was added dropwise to the aqueous phase while stirring at 1500 rpm to form an emulsion, which was further stirred at 1500 rpm for 5 min followed by homogenization at 15,000 rpm for 7 min. The final emulsion was diluted with 20 ml distilled water and stirred at 1000 rpm for 3 h to allow the diffusion and evaporation of organic solvent. NS were recovered by centrifugation at 15000 × g at 4 °C for 30 min. The pellet was suspended in 1 ml water with 5% (w/v) trehalose dihydrate for cryoprotection. The NS were pre-frozen for 8 h at −80 °C and freeze-dried under 0.004 mBar pressure at −50 °C for 24 h followed by secondary drying at 20 °C for 12 h.

For the preparation for CsA loaded NS, the drug (10 mg) was dissolved in the organic phase for 20% (w/w) loading with respect to the polymer. All other steps in the preparation remained the same except the replacement of homogenization with sonication at 20% amplitude for 30 sec and the concentration of DMAB used, in this case 0.25%.

### Characterization of PLGA-GA NS

Particle size of blank and drug loaded NS was detected using Brookhaven dynamic light scattering system (DLS). The sample was prepared by diluting a small amount of fresh NS suspension or freeze-dried sample in 3 ml distilled water.

The morphology and size of the NS was further evaluated using JEOL JSM-7500F ultra-high resolution field emission scanning electron microscope (FE-SEM). The samples for SEM were prepared by depositing 2 μl suspension of freeze-dried NS on 20 mesh copper TEM grids with lacy carbon (Ted Pella Inc.) and subsequent drying. The lacy carbon on the copper grid provides a porous substrate for the nanoparticles to settle without forming an aggregate due to the surface tension of sugary water as on traditional plain SEM substrates. The samples were coated with 4 nm layer of platinum/palladium to introduce conductivity.

Fresh NS suspensions were also subjected to UV spectrophotometry for the evaluation of their surface chemistry. Similar to the polymer solutions, wavelength scans in UV and visible regions were performed for all the NS suspensions. The UV spectra of the NS displayed similar absorbance pattern to polymer blends in solution but were considerably broader due to scattering of light by the motion of NS in suspension.

The surface chemistry of the NS was further evaluated by X-ray photon spectrophotometry (XPS) using Omicron XPS system with magnesium ion source. Survey, C 1 s, O 1s, and N 1s scans of the NS surface were performed on freeze-dried samples deposited on carbon tapes. The freeze-dried NS were smeared on approximately 0.25 mm^2^ sections of carbon tape attached to the sample pucks for XPS and the extra powder was removed using an argon pressurized purge. Equal area of carbon tape used to prepare samples ensured that the same amount of NS powder was deposited in each case. Extra caution was observed with maintaining the dryness of samples as moisture absorbed by trehalose in the NS caused outgassing in the high vacuum environment, thus disrupting sample analysis by shutting off of the system due to vacuum loss. All the characterization protocols had been thoroughly optimized before the final measurements were obtained.

For drug entrapment, the pellets of the CsA loaded NS obtained after centrifugation were dissolved in 3 ml acetonitrile. The amount of drug entrapped in the NS was determined by HPLC with a diode array detector (DAD) (Agilent 1220 infinity), using ODS Hypersil (150 × 4.6 mm; 5 μm) column at 70 °C with acetonitrile: Methanol: 0.05 M Phosphoric acid (45:40:15) as mobile phase eluting at 0.7 ml/min. The wavelength of the DAD was set to 210 nm with 360 nm reference wavelength and a window of ± 4 nm. The retention time of CsA was 5.3 min. The percent entrapment efficiency of CsA in the NS was calculated using equation (1).


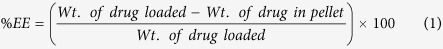


### ELISA study to evaluate the non-competitive binding of PLGA-GA NS to TfR

All variations of fluorescent and non-fluorescent PLGA-GA/PLGA NS were suspended in dH_2_O at the concentration of 250 μg/ml. 25 μl of each NS suspension was added to the wells of 96-well ELISA plate. All experiments were performed in triplicates. At these concentrations, the amount of GA available on the NS in each 25 μl sample amounts to 0, 50, 150 and 250 ng/ml for PLGA, PLGA-GA20, PLGA-GA60 and PLGA-GA100 respectively. To study the binding affinity of PLGA-GA NS to TfR, 25 μl of 200 ng/ml solution of TfR and 150 μl of horseradish peroxidase (HRP)-conjugated 2° antibody were added into each well containing samples. The samples were mixed for 10 min on a rocker. The reaction was allowed to proceed for another two hours without shaking at room temperature. ELISA plate was then inverted to empty the contents of the wells. At this point, the fluorescence in the wells with F-NS samples was measured using microplate reader at excitation/emission (Ex/Em) specific to F-NS (552/575 nm) to quantify the total F-NS available in each well. The wells were washed three times with the washing buffer provided in the ELISA kit and dried with paper towels. 200 μl of substrate solution, also provided with the ELISA kit, was added to the wells and incubated in the dark at room temperature for 30 min. To quantify TfR, the absorbance optical density (OD) of each well was measured at 450 nm using a microplate reader with background correction wavelength of 570 nm. Fluorescence was also measured again for the F-NS sample wells to quantify bound NS.

In the second study, 7-peptide, a TfR specific ligand, was used in an attempt to block the active site of TfR to assess the non-competitive binding of PLGA-GA NS. A 25 μl of 250 ng/ml solution of TfR with 2.5 μl of 25, 75 or 150 ng/ml of 7-peptide solution were added into the wells. 150 μl of HRP-conjugated 2° antibody was then added into each well and the reaction was carried as described above and the OD was measured to quantify TfR levels.

Subsequently, blocking experiments were conducted following the similar protocol, where PLGA-GA or PLGA NS were introduced prior to the addition of 2° antibody. In this case, 25 μl of F-NS suspension, 25 μl of 250 ng/ml solution of TfR, 2.5 μl of 150 ng/ml solution of 7-peptide and 150 μl of HRP-conjugated 2° antibody was added into the ELISA plate wells. All other procedures including reaction, washing and analysis remained the same as described for the first ELISA study.

### Caco-2 cell culture

Caco-2 cells (passage 7) were seeded at a density of 4.0 × 10^5 ^cells/well in a 6-well plate for all experiments (n = 3) and cultured for 48 h in 2 ml Dulbecco’s Modified Eagle Medium (DMEM/F-12) containing 10% fetal bovine serum (FBS) and 1% Penicillin/Streptomycin solution (v/v) at 37 °C/5% CO_2_.

### Tuning avidity of PLGA-GA NS to TfR in caco-2 cells

Freeze-dried F-NS were suspended in dH20 at a concentration of 250 and 500 μg/ml. Caco-2 cells were incubated with the 200 μl of F-NS suspension for 5 min at 37 °C/5% CO_2_, followed by addition 1.8 ml serum free medium and further incubation for 1 h at 37 °C/5% CO_2_. The cells were washed three times with phosphate buffered saline (PBS, pH 7.4) and further processed for fluorescent microscopy and FACS as described below. No noticeable changes were observed in the cell morphology or size due to addition of 200 μl of water (SI Figure 10). There were reports indicating that 25% hypotonic stimulus caused either little or no swelling in caco-2 cells due to the lack of caveolin-1, a 21–24 kDa membrane protein that modulates the activity of the volume-regulated anion channels (VRACs)^44^,^45^,^46^. In this study, the volume of water used constitutes ~10% of total volume and the cells stayed in contact with water only for 5 min.

### Uptake of PLGA-GA NS across caco-2 cells after blocking TfR

For blocking experiments, caco-2 cells (n = 3) were incubated with 10 μg/mL of human/mouse specific TfR primary antibody in 6-well plate for 30 min at 37 °C in CO_2_ incubator. After three consecutive washings with PBS, F-NS were added and incubated for further 1 h. These were then washed with PBS 3 times and trypsinized and subjected to FACS analysis.

### Uptake of PLGA-GA NS in caco-2 cells after pretreatment with GA

For the receptor blocking experiment, caco-2 cells (n = 3) were pre-incubated with 10, 20 and 30 μM GA at 37 °C/5% CO_2_ for 15 min prior to the addition of F-NS. 5 mM GA stock solution was previously prepared in dimethyl sulfoxide (DMSO) and 2, 4 and 6 μl was added to cell culture medium to obtain 10, 20 and 30 μM (6.29; 12.58; 18.86 μg) final concentration respectively. After incubation with F-NS, the cells were washed three times with PBS and further processed for fluorescent microscopy and FACS as described below.

### Concentration dependent toxicity in caco-2 cells

In order to study the effect of increasing concentration of GA on the viability of caco-2 cells (n = 3), they were incubated with 10, 20 and 30 μM molecular GA for 15 min at 37 °C/5% CO_2_ and washed with PBS. In addition, cells were treated with all variations PLGA-GA/PLGA NS in the concentration of 250 μg (5 μg GA) and 500 μg (10 μg GA)/ml for 1 h at 37 °C/5% CO_2_ and subsequently washed with PBS and further processed for FACS analysis as described below.

### Fluorescent Microscopy

The cells were fixed by keeping them in 4% paraformaldehyde for 20 min at room temperature and subsequently washing three times with PBS. The fixed cells were stained for actin (green) and nuclei (blue) using ActinGreen and NucBlue molecular probes. The cells were imaged using EVOS-FL microscope at 60× magnification.

### Fluorescence activated cell sorting/scanning (FACS)

The washed cells were dissociated using 0.25% trypsin/0.02% (Ethyelendiaminetetraacetic acid) EDTA solution to obtain a cell suspension which was centrifuged at 400 × g/4 °C for 5 min. The cells were re-suspended in 500 μl PBS containing 1% (w/v) bovine serum albumin (BSA) and centrifuged at 400 × g/4 °C for 5 min. This washing step was repeated 3 times. The final cell suspension in PBS with BSA was subjected to FACS analysis using BD FACS Calibur instrument. FACS data was analyzed using Flow Jo 9.8.5 software.

### Animals

Male SD rats (210–230 g) (Harlan, USA) colony was maintained in the animal quarterin accordance to the Texas A&M Institutional Animal Care and Use Committee (IACUC).

### Animal Study Approval

All experimental protocols were reviewed and approved by the Texas A&M University IACUC. All animal procedures for this study were carried out in accordance with the relevant IACUC guidelines and approved protocols.

### *Ex vivo* transport of PLGA-GA NS through intestinal barrier

Male SD rats (n = 3) (210–230 g) were euthanized by CO_2_ asphyxiation followed by cervical dislocation. The jejunum regions of the small intestine of SD rats were dissected, washed with ice cold PBS three times and cut into 2 inch long pieces. One end of each truncated small intestine section was tied with a nylon thread and F-NS at the concentration of 250 μg/ml in dH_2_O were pipetted into the open end. After adding F-NS, the open end was closed with fine forceps and tied with nylon thread. This bag-like structure was immersed in Hank’s Balanced Salt Solution (HBSS) with calcium and magnesium, in a 30 mm petri dish and incubated at 37 °C/5% CO_2_, for 2 h with intermittent shaking at 15 min intervals. After 2 h, small intestine section was removed from the dish, the ends were clipped and the open tube thus obtained was washed with the PBS three times to remove unbound F-NS. The small intestine tissue samples were embedded in Optimal Cutting Temperature (OCT) media for sectioning and cut into 4-μm thick cross-sections with a cryostat (ThermoScientific, USA) at −20 °C. The tissue sections were further processed for confocal imaging as described in later sections.

### Kinetics of fluorescent PLGA-GA NS

Male SD rats (n = 3) were perorally dosed 50 mg/kg (~16 mg/rat) PLGA or PLGA-GA100 (~325 μg GA) suspended in 1 ml water using an oral gavage needle. The rats were allowed to have access to standard chow and water for the duration of study. Animals were sacrificed at 2, 12 and 24 h by CO_2_ asphyxiation followed by cervical dislocation. Blood (~5 mL) was collected from the sacrificed animals via heart puncture. In addition, small intestine (jejunum ~5 cm), liver, spleen, kidneys, and brain were excised for confocal imaging described in the later section. Small intestines’ were flushed with saline several times at room temperature to remove unabsorbed NS and food particles.

Blood smears were prepared using fresh blood (2 μl/coverslip) and imaged using EVOS-FL microscope at 60× magnification in differential interference contrast (DIC) and fluorescence imaging modes for particles and both were later merged to get final images. Fluorescence was quantified using image J software (NIH). Plasma was separated from blood by centrifugation at 1620 × g/4 °C for 20 min. Plasma fluorescence was observed in Infinite F200 microplate reader (Tecan) at Ex/Em specific for F-NS (552/575 nm) in samples diluted to 1:50 in PBS.

### Confocal microscopy

#### Caco-2 cells

50,000 Caco-2 cells/mL were cultured for 48 h at 37 °C and 5%CO_2_ according to manufacturer’s protocol on 8 well 1 μm-slide ibiTreat (ibidi, 82152 Martinsried, Germany) culture plate. Cellular uptake experiment was performed as previously mentioned for cell treatment with 3 μg/ml F-NS. Images were acquired on CLSM at 40× original magnification. Images were processed using Zen 2012 software.

#### Ex vivo intestinal sections

For imaging the cryosections of rat jejunum from the ex-vivo experiment, they were stained with primary mouse monoclonal anti-TfR-CD71 antibody diluted 1:200 in PBS containing 3% goat serum and incubated overnight at 4 °C. The sections were then washed with PBS and anti-mouse secondary antibody conjugated with Alexa Fluor 488 was added. The sections were further incubated at room temperature for 2 h and washed three times with PBS. Nuclei were stained with DAPI.

#### Tissue sections from in vivo fluorescent NS study

To image the tissue section from small intestine, liver, spleen, kidney and brain from rats from *in vivo* F-NS study were also embedded in OCT medium and 4 μm cryosections were prepared using cryostat. All samples were stored at −80 °C until further processing. DAPI (Prolong Gold antifade mountant with DAPI) was used to stain the nuclei in all tissue sections. Similar to the *ex vivo* study, the small intestine sections from the *in vivo* study were stained for TfR using primary and secondary antibodies.

Multiple optical sections of each tissue sample were imaged using a ZEISS LSM 780 confocal laser scanning microscope. Images captured during microscopy analysis of the tissue sections were analyzed using ZEN software. Four images were captured for each animal in a group for each tissue (total of 12 images per group). Image J software (NIH) was used to measure and compare the fluorescence intensity of the tissue samples.

### Cyclosporine A (CsA) kinetics

Plain CsA (15 mg/kg in carboxymethylcelluose), PLGA and PLGA-GA100 NS (~41 mg for 15 mg/kg CsA based on 45% EE at 20% loading) suspended in 1 ml water were dosed perorally to the respective groups of rats (n = 4) using an oral gavage needle. Blood samples (100 μl) were collected from rats through tail vein at 0.5, 1, 2, 6, 12, 24, 48 and 72 h and stored in BD microtainer tubes containing K_2_EDTA as anticoagulant and stored at 4 °C. Blood samples were centrifuged at 1620 × g/4 °C for 20 min to separate plasma which was then stored at −80 °C. CsA concentration in plasma was measured using CsA ELISA kit specific for rat. Plasma was diluted for the CsA analysis (8.5 times) with the diluent provided in the kit. All rats were sacrificed at 72 h by CO_2_ asphyxiation followed by cervical dislocation. Liver and brain tissues were homogenized in tissue lysis buffer and centrifuged at 20817 × g/4 °C for 30 min. Supernatant was collected and stored at −80 °C. CsA ELISA kit was used to measure tissue level concentrations of CsA after appropriate dilution (5 times in this case) using the diluent provided by the manufacturer and converted in to the μg/gram weight for each tissue.

### Statistical analysis

All values are expressed as mean ± standard error of the mean (SEM), and *p*-values were assessed by one-way analysis of variance (ANOVA). For the kinetics study two-way ANOVA was used. A *p* < 0.05 was considered statistically significant. Comparison of more than two groups (PLGA and PLGA-GA NS and between the groups) was performed with Tukey’s multiple comparison tests using Prism version 5 (GraphPad Software).

## Additional Information

**How to cite this article**: Saini, P. *et al*. The Next Generation Non-competitive Active Polyester Nanosystems for Transferrin Receptor-mediated Peroral Transport Utilizing Gambogic Acid as a Ligand. *Sci. Rep*. **6**, 29501; doi: 10.1038/srep29501 (2016).

## Supplementary Material

Supplementary Information
