# Supplementary material for: The Next Generation Non-competitive Active Polyester Nanosystems for Transferrin Receptor-mediated Peroral Transport Utilizing Gambogic Acid as a Ligand
Source: Sci Rep. 2016 Jul 8;6:29501. doi: 10.1038/srep29501 (PMC4937428; doi:10.1038/srep29501)
Supplement: Supplementary Information [file srep29501-s1.pdf]

Supplementary Information

The Next Generation Non-competitive Active Polyester Nanosystems for Transferrin Receptor-mediated Peroral Transport Utilizing Gambogic Acid as a Ligand[$]

P. Saini+, R. Ganugula+, M. Arora and M. N. V. Ravi Kumar*

Department of Pharmaceutical Sciences

Texas A&M Health Science Center

Reynolds Medical Building, Mail Stop 1114

College Station 77843, Texas

+Authors contributed equally; *Author for correspondence E-mail: mnvrkumar@tamhsc.edu

Phone: +1-979-436-0721

[$]Part of the work presented in this manuscript has been accepted for *Research Highlight Talk with a corresponding Poster, in "Taking Stock of Progress and Challenges in Drug Delivery and Targeting session" at the 43rd Annual Meeting & Exposition of the Controlled Release Society, July 17-20, 2016.*

Table of Contents

1 Characterization of PLGA-GA…………………………………………………………………… 2
2 Characterization of PLGA-GA NS………………………………………………………………… 3
3 Stability of PLGA-GA and its NS in transit conditions…………………………………………… 4
4 ELISA study to evaluate the non-competitive binding of PLGA-GA NS to TfR……………………………………………………………………………………………… 5
5 Transport studies of PLGA-GA NS with/without blocking of TfR and toxicity evaluation in cultured cells………………………………………………………………………… 8
5.1 Tuning avidity of PLGA-GA NS to TfR in caco-2 cells……………………………………… 8
5.2 Uptake of PLGA-GA NS in caco-2 cells after pre-treatment with GA………………… 10
6 *Ex vivo* transport of PLGA-GA NS through intestinal barrier……………………………… 11
7 Kinetics of fluorescent PLGA-GA NS…………………………………………………………… 13
8 References…………………………………………………………………………………………… 18

1. Characterization of PLGA-GA

PLGA-GA were subjected to GPC and MALDI analyses to evaluate the effect of conjugation process on the molecular integrity of the polymer (Figure S1a,b). Both the techniques reflect that the molecular weight distribution did not alter after the conjugation of GA to PLGA suggesting the preservation of polymer backbone. The thermal properties of PLGA were conserved during the GA conjugation process, as the glass transition temperature of the end polymer did not exhibit any substantial changes in the deflection point in DSC when compared to the parent polymer (Figure S1c). PLGA-GA20/60 exhibit much higher contact angle compared to PLGA-GA100, but significantly lower to PLGA (Figure S1d).

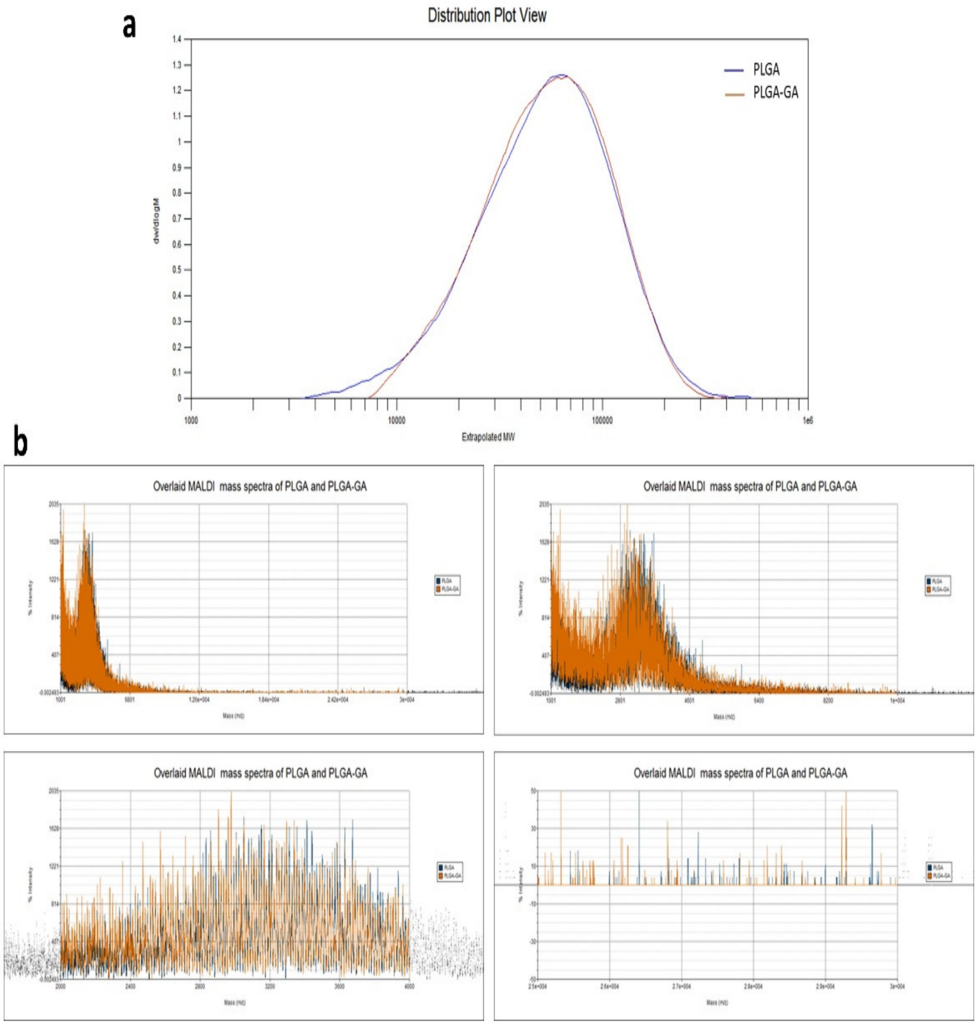

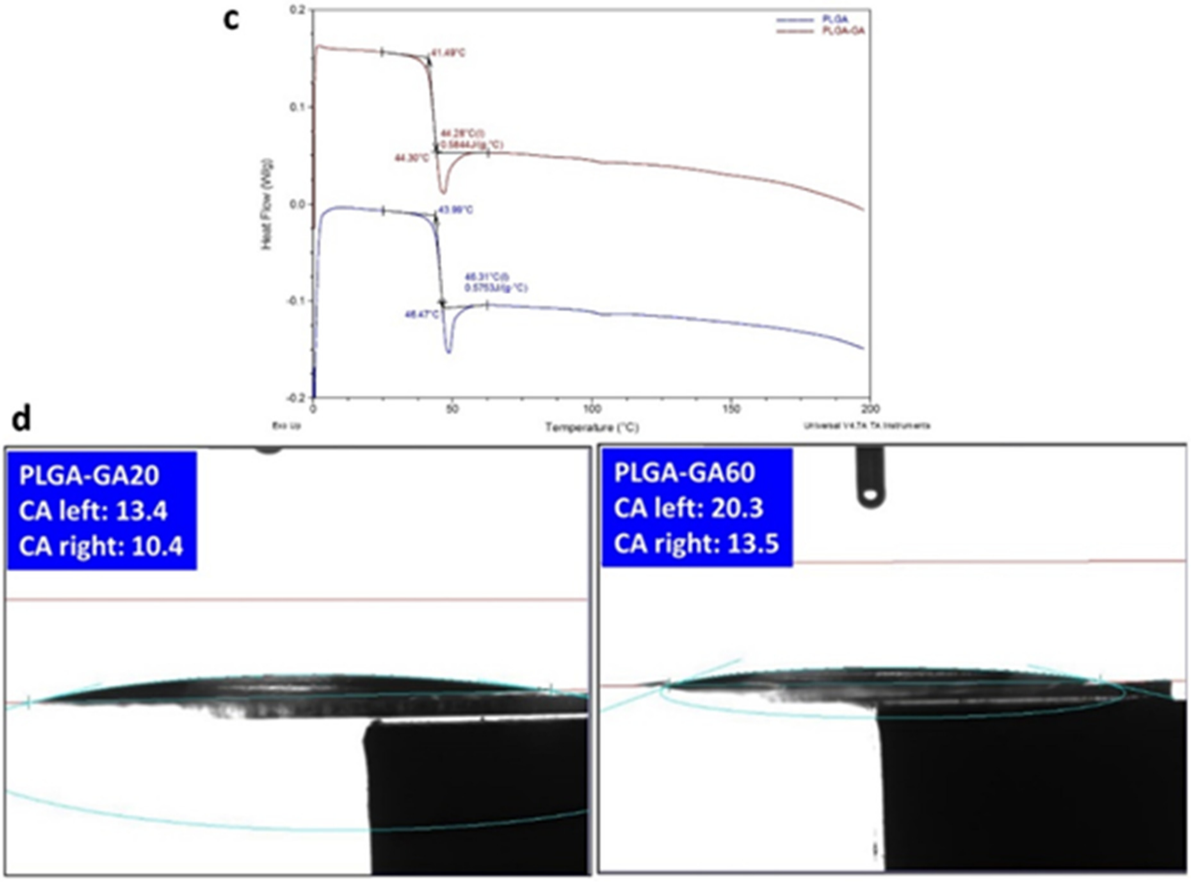

Figure S1. a, GPC chromatogram showing similar molecular weight distribution for PLGA and PLGA-GA. **b,** MALDI spectra of PLGA and PLGA-GA. **c,** DSC thermograms of PLGA and PLGA-GA. **d,** Contact angle of water droplets on PLGA-GA20, PLGA-GA60 polymer coated glass slides.

2. Characterization of PLGA-GA NS

All the PLGA-GA/PLGA NS variations were analyzed using DLS and SEM for their size profile and morphology. The zeta potential for both blank and drug loaded particles were ~25-30 mV at pH ~6. Figure S2 presents the DLS size distribution and SEM micrographs of PLGA, PLGA-GA20 and PLGA-GA60 NS that were analyzed in addition to PLGA-GA100 NS presented in the main body of the paper.

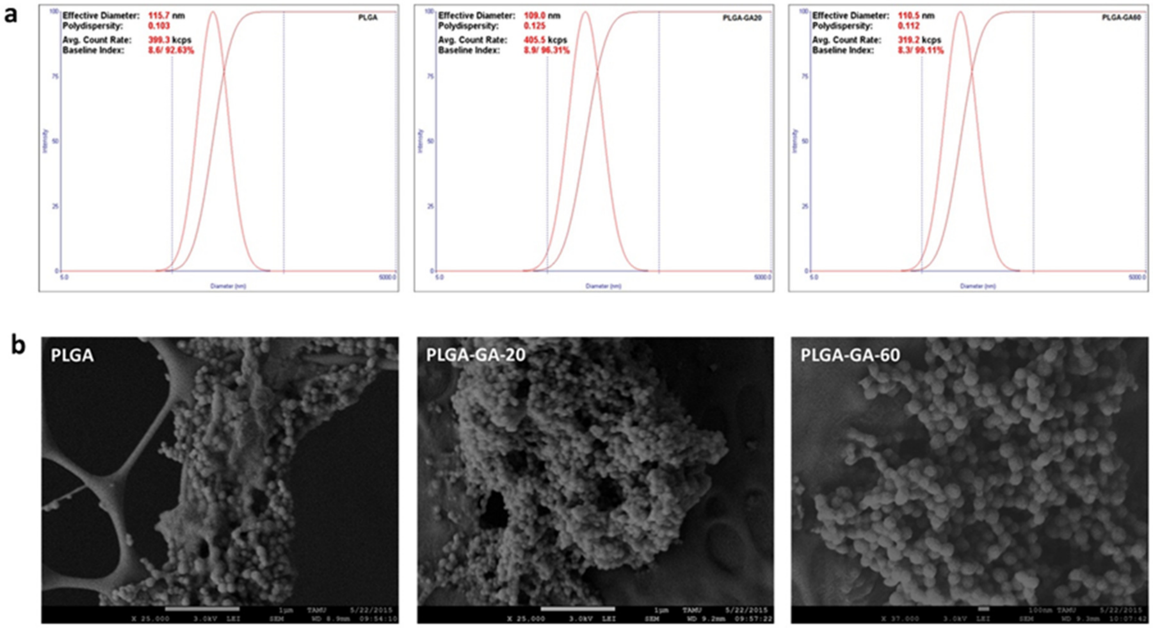

Figure S2. Size profile and morphology analysis data for PLGA, PLGA-GA20 and PLGA-GA60 corresponding to the scheme presented in Figure 1c in the main body of the paper. **a,** DLS size distribution plots of PLGA. **b,** SEM micrographs of PLGA-GA NS variations.

3. Stability of PLGA-GA and its NS in transit conditions

Peroral dosage of PLGA NS has been previously reported by our group[1] where the NS were stable in gastrointestinal (GI) conditions. The stability of PLGA-GA in acidic conditions was evaluated by dissolving the polymer in 0.01N HCl solution in ethyl acetate (pH 2) and monitoring its absorbance at 290 nm in a UV/Vis spectrophotometer for 2 h. Wavelength scan spectra of the polymer in solution were also obtained at the beginning and end of the study for comparison among them and with the spectrum of free GA in solution. The spectra of the polymer at the beginning and at the end of the study were similar and no

shift in the peaks was observed to indicate the cleavage of GA from the polymer (Figure S3a).

In addition to evaluating the chemical stability of the polymer in acidic conditions, the stability of PLGA-GA NS was studied in suspension at pH 2 and 7,4 at 37°C for 2 and 6 h respectively in a shaking water bath. At the end of the incubation period for each sample, the size profile of the NS was analyzed using DLS. The NS subjected to acidic condition displayed a size of 159 nm with a PDI of 0.197 while those at pH 7.4 in PBS were 564 nm with a PDI of 0.333 (Figure S3b,c). The larger size observed by DLS could be the result of aggregation discussed later in section 5.1.

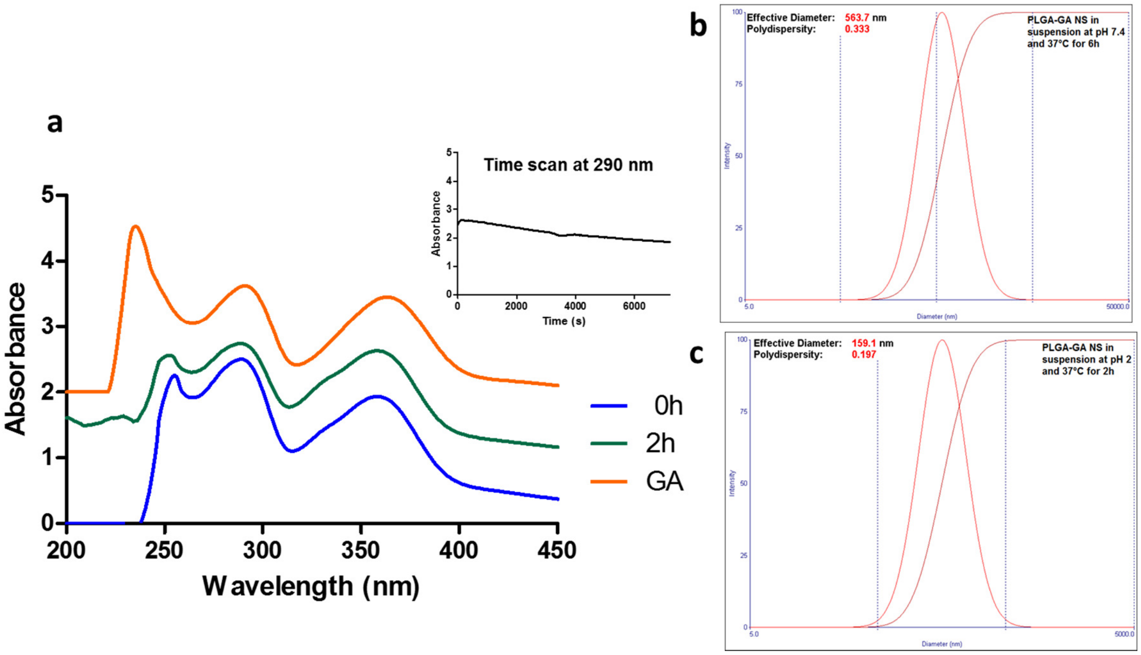

Figure S3. a, UV/Vis spectra of PLGA-GA in 0.01N HCl solution in ethyl acetate at 0 and 2 h with the spectrum of free GA. The inset shows the absorbance of PLGA-GA in acidic conditions at 290 nm for the duration of the study. **b/c,** DLS size profile plots of PLGA-GA NS in suspension at pH 2 and 7.4 respectively.

4. ELISA study to evaluate the non-competitive binding of PLGA-GA NS to TfR

As mentioned in the main communication, the total fluorescence intensity (FI) of the fluorescent NS (F-NS) in the ELISA wells was measures before washing. This aided in the confirmation that all wells were subjected to equal amount of NS and the final FI after the washing of wells was due to the binding of NS to TfR. A colorimetric assay using a 2° TfR antibody was also performed to detect the amount of free TfR after the addition of non-fluorescent PLGA-GA/PLGA NS. As in the case of F-NS presented in the main communication, the addition of non-fluorescent NS to TfR did not cause any decline in the levels of free TfR (Figure S4a-f).

Since a small aliquot of NS suspension of 25 µl was employed in the ELISA experiment after a dilution of ~4000x from the initial 250µg/ml suspension, it was imperative to confirm

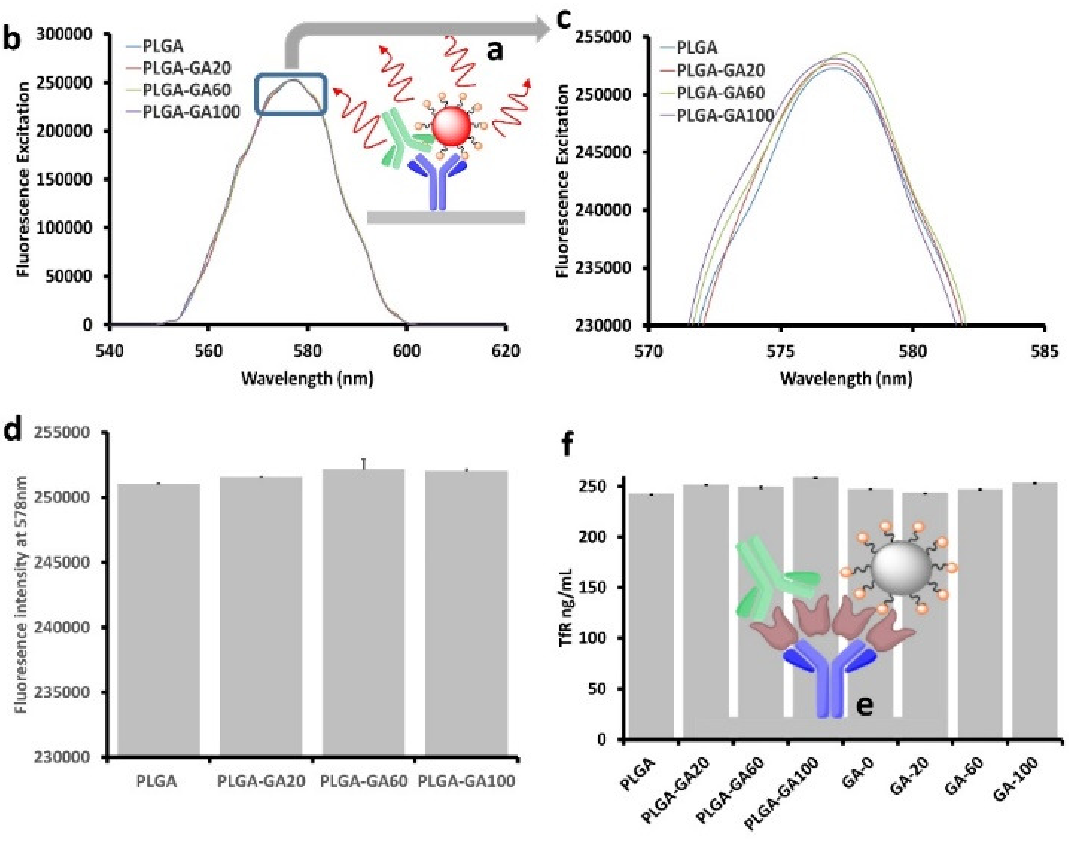

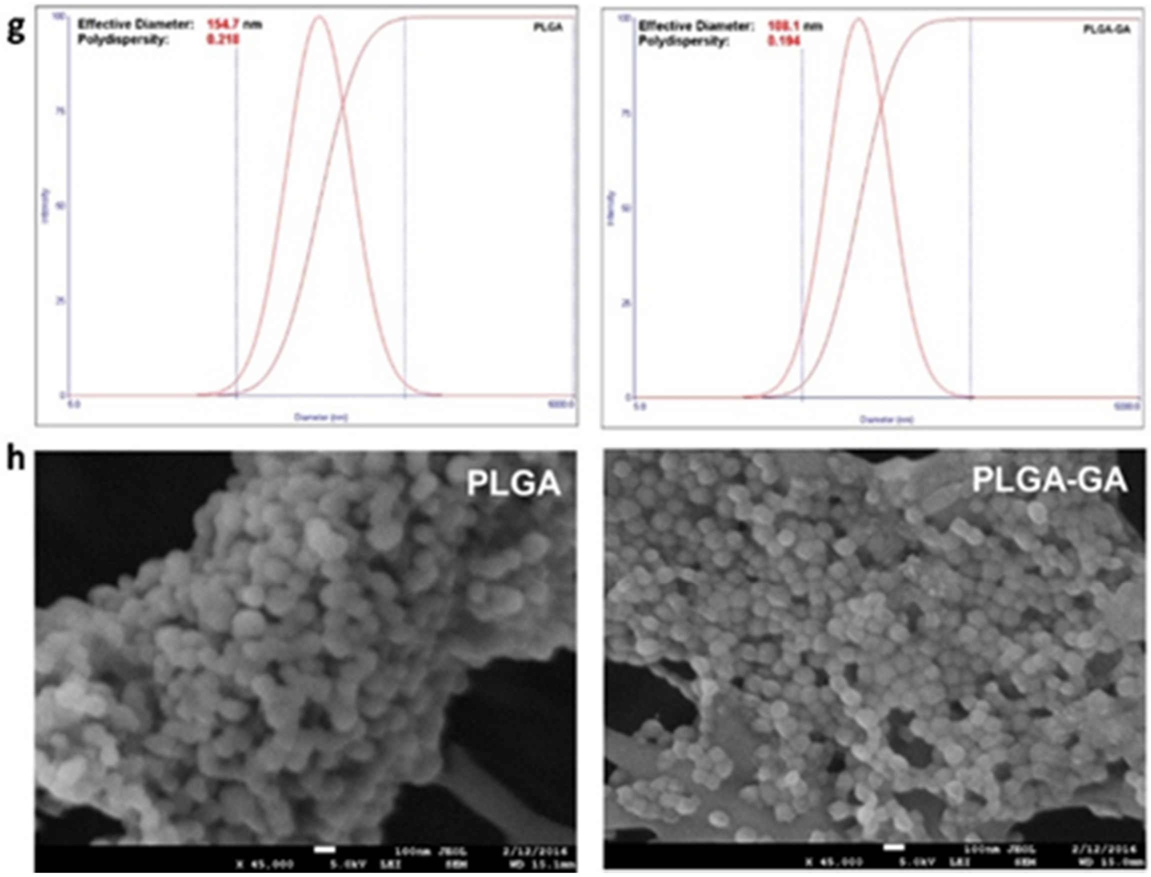

the presence of NS in the aliquot. DLS and SEM analysis of the 25µl aliquots confirmed the presence of adequate NS in the samples (Figure S4g,h).

Figure S4. a, Illustration of the initial study involving binding of fluorescent PLGA-GA/PLGA NS to 1º antibody followed by addition of 2º antibody (No TfR). **b,** Wavelength scan of total fluorescence of the same volume NS suspensions in the well without TfR. **c,** Enlarged image of the florescence spectra at the λ_{max} for rhodamine (578 nm). **d,** Histogram of total fluorescence of entire volume of NS suspensions in the well. **e,** Illustration of experimental design to study the effect of bound PLGA NS, PLGA-GA NS or GA followed by addition of 2º antibody on TfR levels measured by colorimetric assay. **f,** TfR levels showing no decrease from the initial levels on binding with PLGA-GA/PLGA NS and free GA. **g/h,** The DLS size distribution plots and SEM micrographs of PLGA or PLGA-GA100 NS at the dilutions used in the ELISA study.

5. Transport studies of PLGA-GA NS with/without blocking of TfR and toxicity evaluation in cultured cells

5.1 Tuning avidity of PLGA-GA NS to TfR in caco-2 cells

It was observed in the trail experiments before the final experimental set up for cellular uptake that the NS agglomerated when suspended in cell culture media. SEM images were obtained for the NS suspended in media showing considerable aggregation (Figure S5). However, such agglomeration was not observed *in vivo* upon peroral dosing in the current study as well as our previous studies on peroral or intravenous dosing[1]. It appears that such agglomeration *in vitro* is neither exceptional nor unusual, but just goes undocumented. The most widely used PLGA NS seems to form larger aggregates compared to PLGA-GA NS, perhaps due to higher hydrophobicity of the PLGA compared to PLGA-GA.

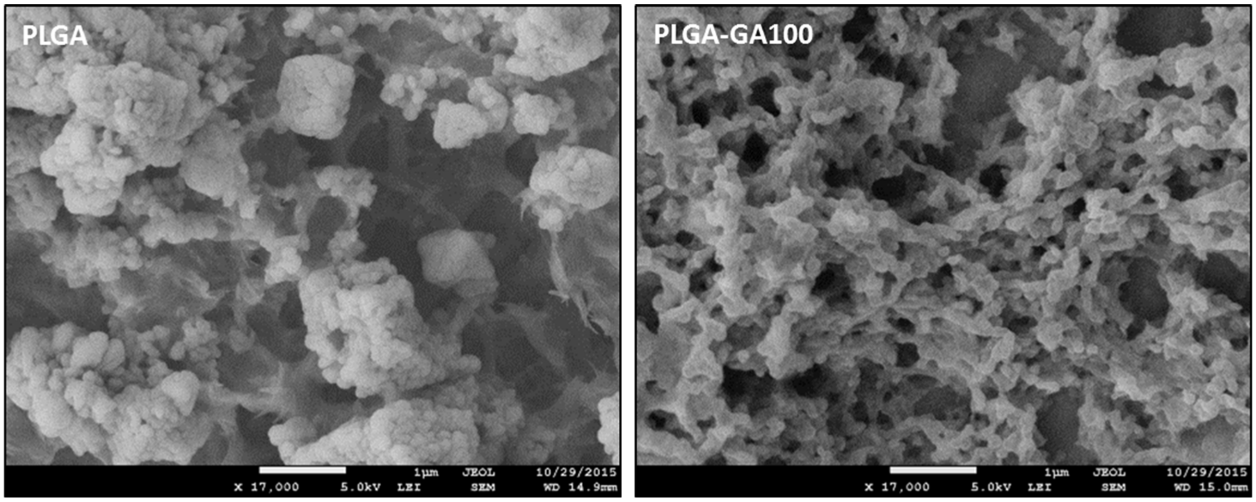

Figure S5. SEM images showing agglomerated PLGA and PLGA-GA100 NS in HBSS media. As mentioned in the main communication, the cellular uptake experiments were performed with two concentrations of NS suspension, 250 µg and 500µg. The fluorescence microscope images and FACS plots for the cells treated with 250 µg NS are presented in Figure S6.

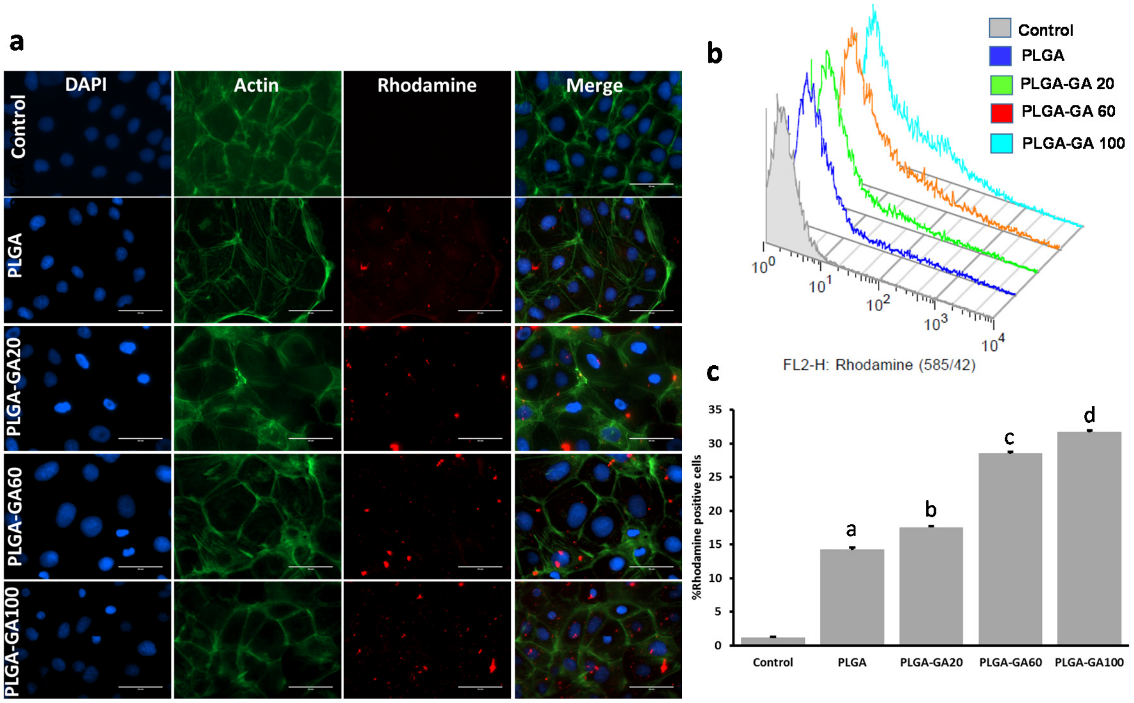

Figure S6. a, Cellular uptake of PLGA-GA/PLGA NS (treated with 250 μg FL-NS) (Red) with cytoskeleton and nuclei labeled Green and DAPI respectively and imaged using EVOS-FL microscope (60X, scale bar represents 50 μm). **b,** FACS analysis of caco-2 cells. **c,** Quantification of rhodamine positive cells *(a vs b,c,d; b vs c,d; c vs d ***p<0.001)*.

5.2 Uptake of PLGA-GA NS in caco-2 cells after pre-treatment with GA

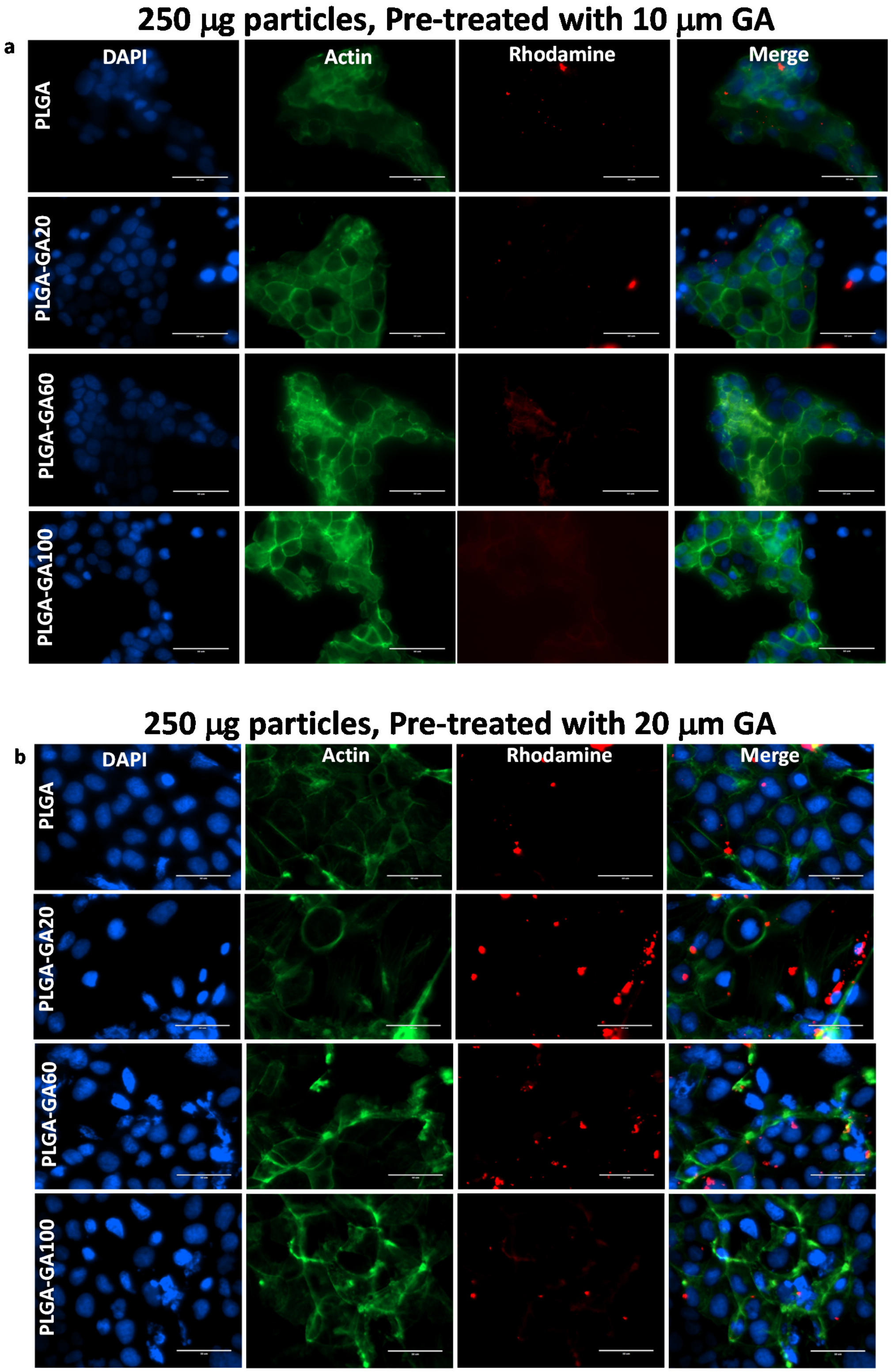

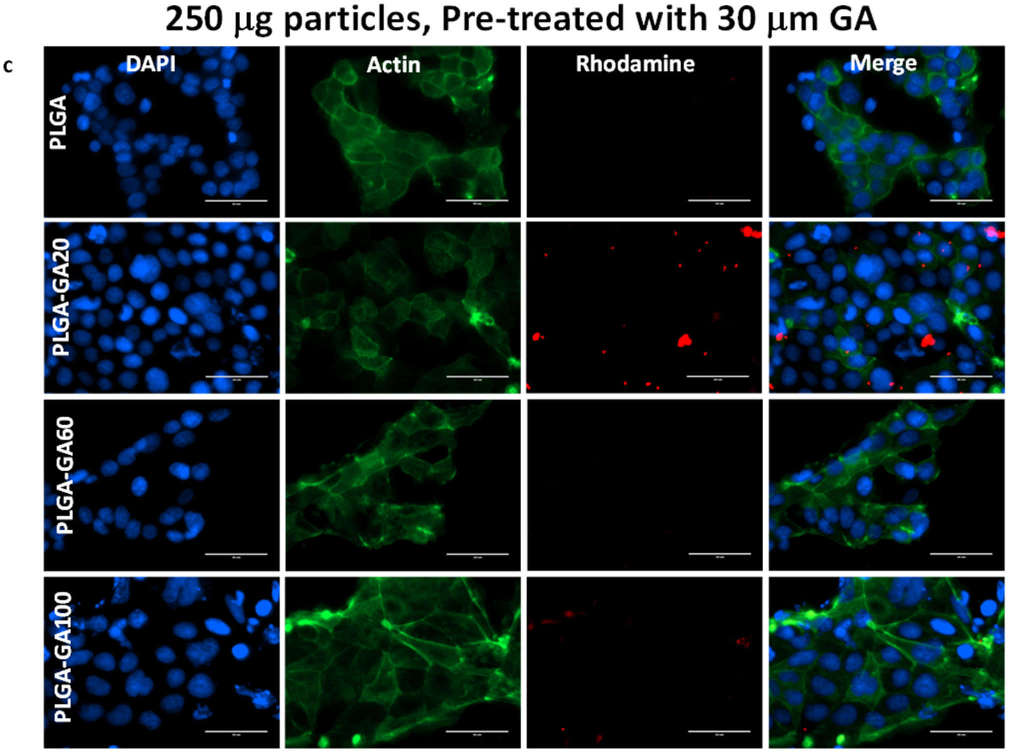

Figure S7. a-c, Representative fluorescent microscope images of cellular uptake of PLGA-GA/PLGA NS uptake in Caco-2 cells treated with 250 µg NS after pre-treatment with 10 µM (a), 20 µM (b) and 30 µM (c) GA. (60X, scale bar represents 50 µm)

6. *Ex vivo* transport of PLGA-GA NS through intestinal barrier

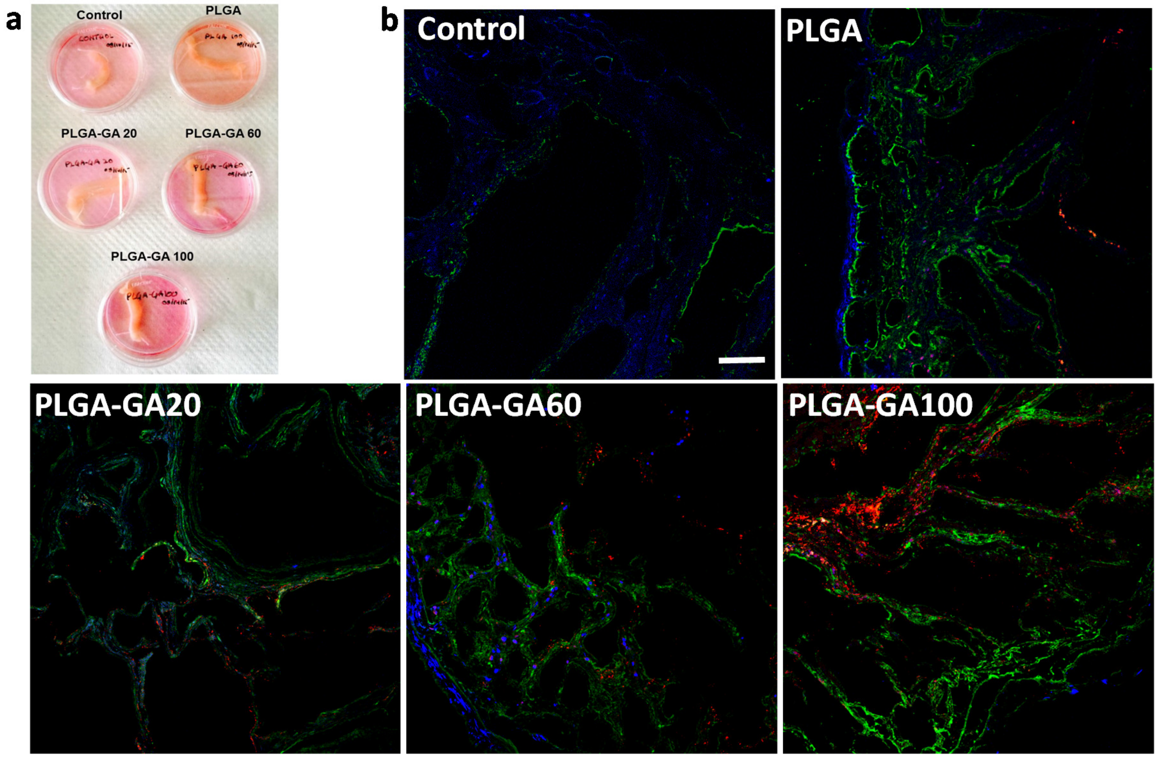

c

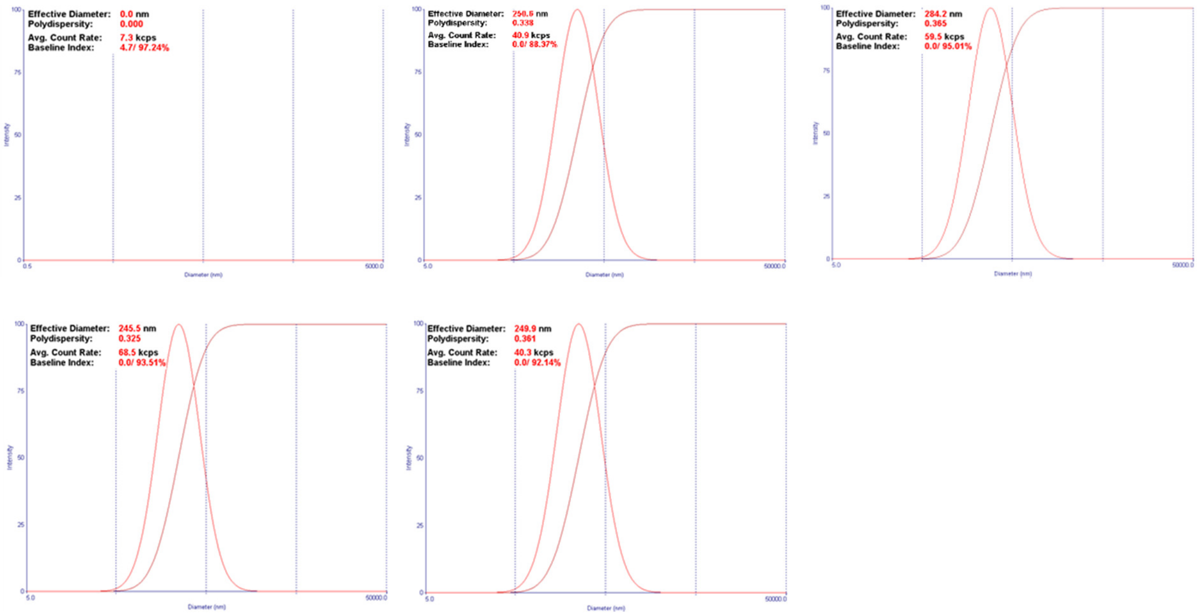

d

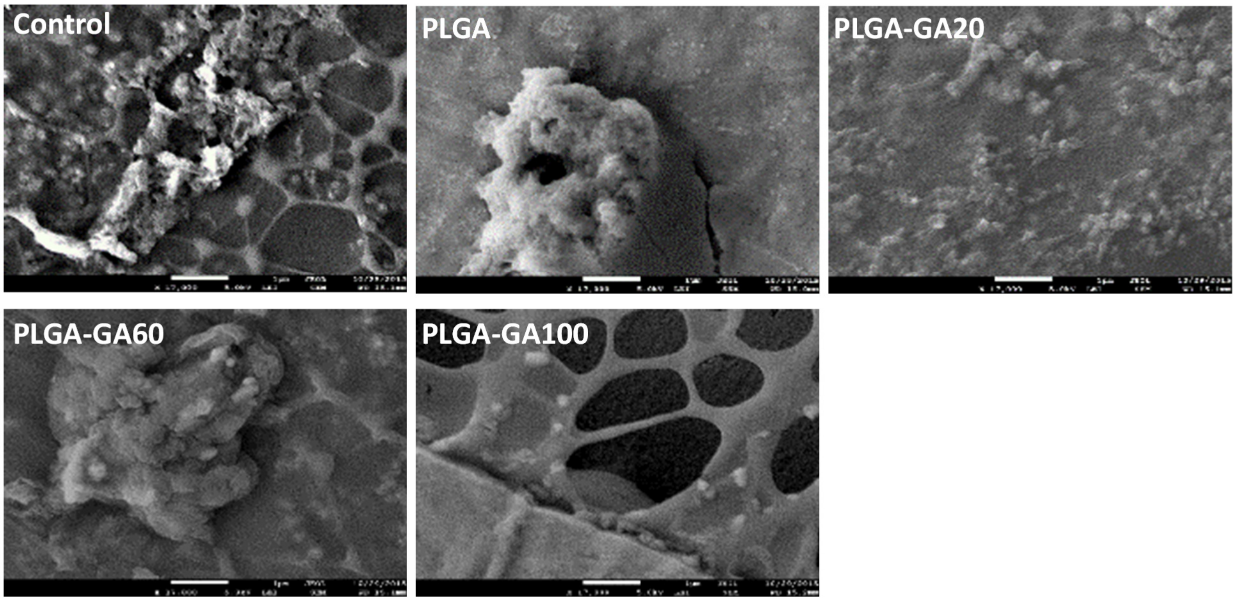

Figure S8. a, Photograph of experimental set-up for *ex vivo* transport of PLGA-GA NS across intestinal barrier in a tubular section of jejunum. **b,** Image of the *ex vivo* intestine sections presented in Figure 3b in main body of the paper with additional labeling of nuclei (blue) with DAPI (12x, scale bar represents 50 μm). **c,** DLS size profile of particles in media at the completion of the transport study at 2 h. **d,** SEM images of freeze dried media at 2 h.

7. Kinetics of fluorescent PLGA-GA NS

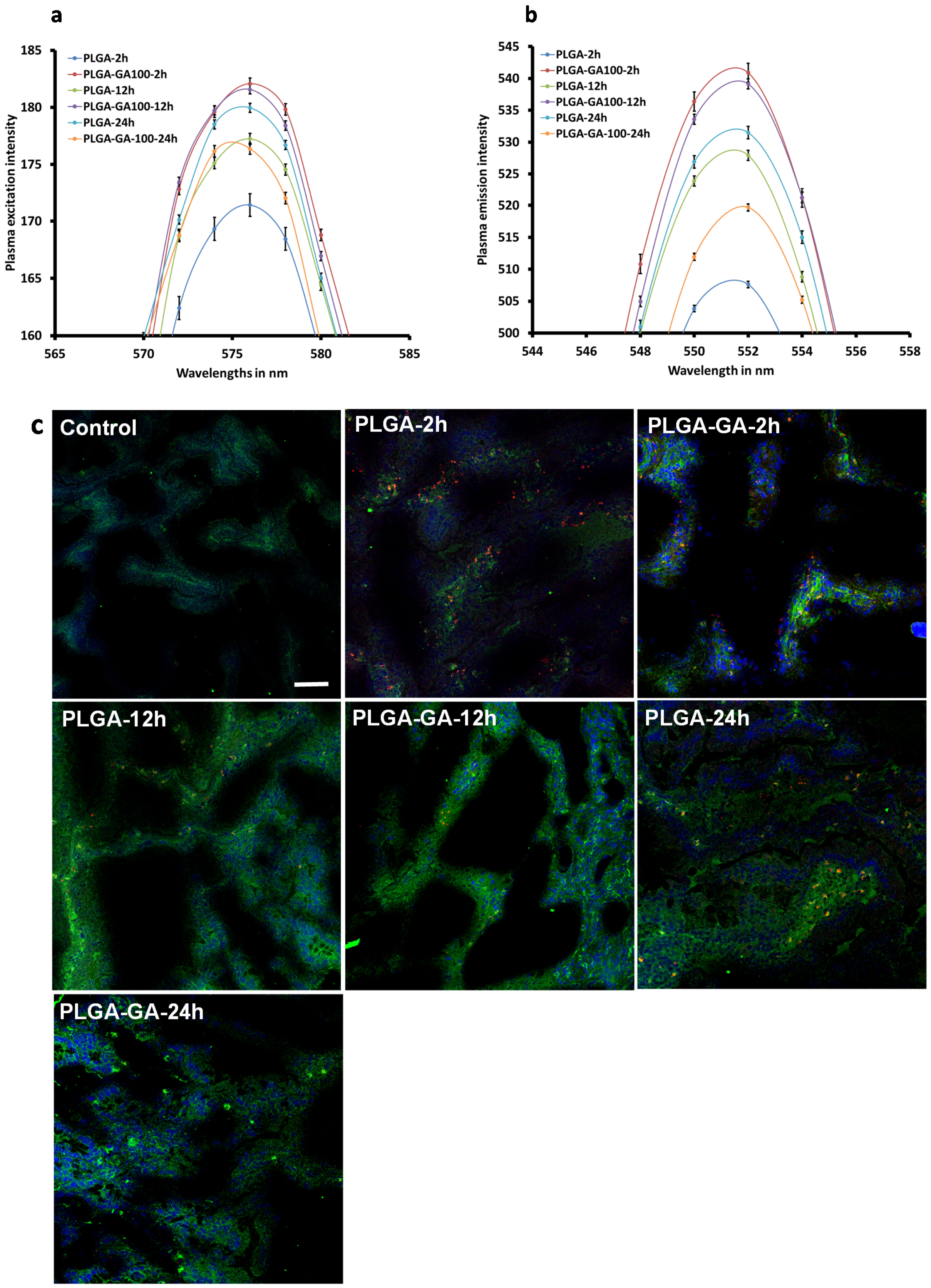

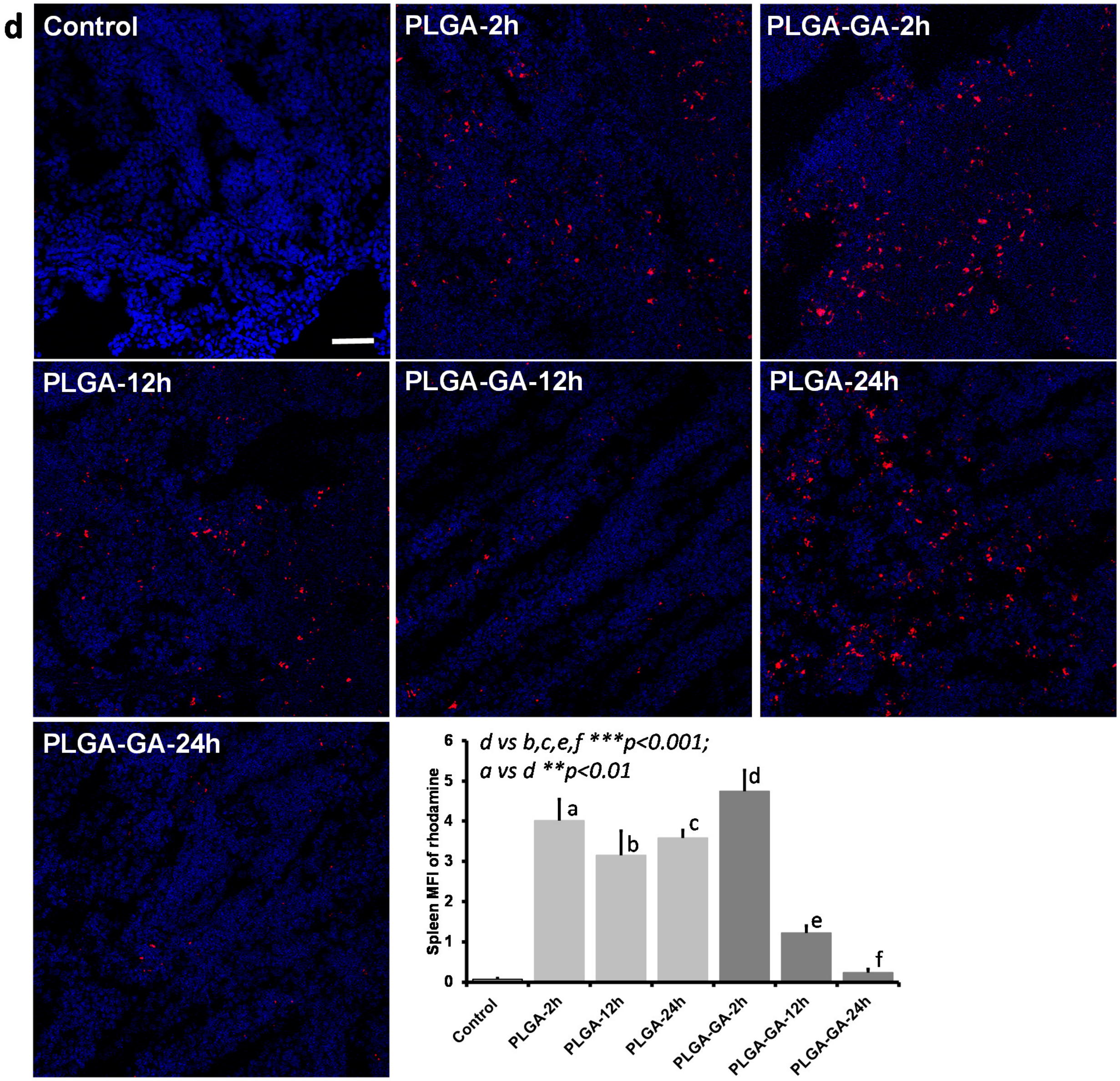
d
Control
PLGA-2h
PLGA-GA-2h
PLGA-12h
PLGA-GA-12h
PLGA-24h
PLGA-GA-24h
d vs b,c,e,f ***p<0.001;
a vs d **p<0.01
Spleen MFI of rhodamine
a
b
c
d
e
f
Control
PLGA-2h
PLGA-12h
PLGA-24h
PLGA-GA-2h
PLGA-GA-12h
PLGA-GA-24h

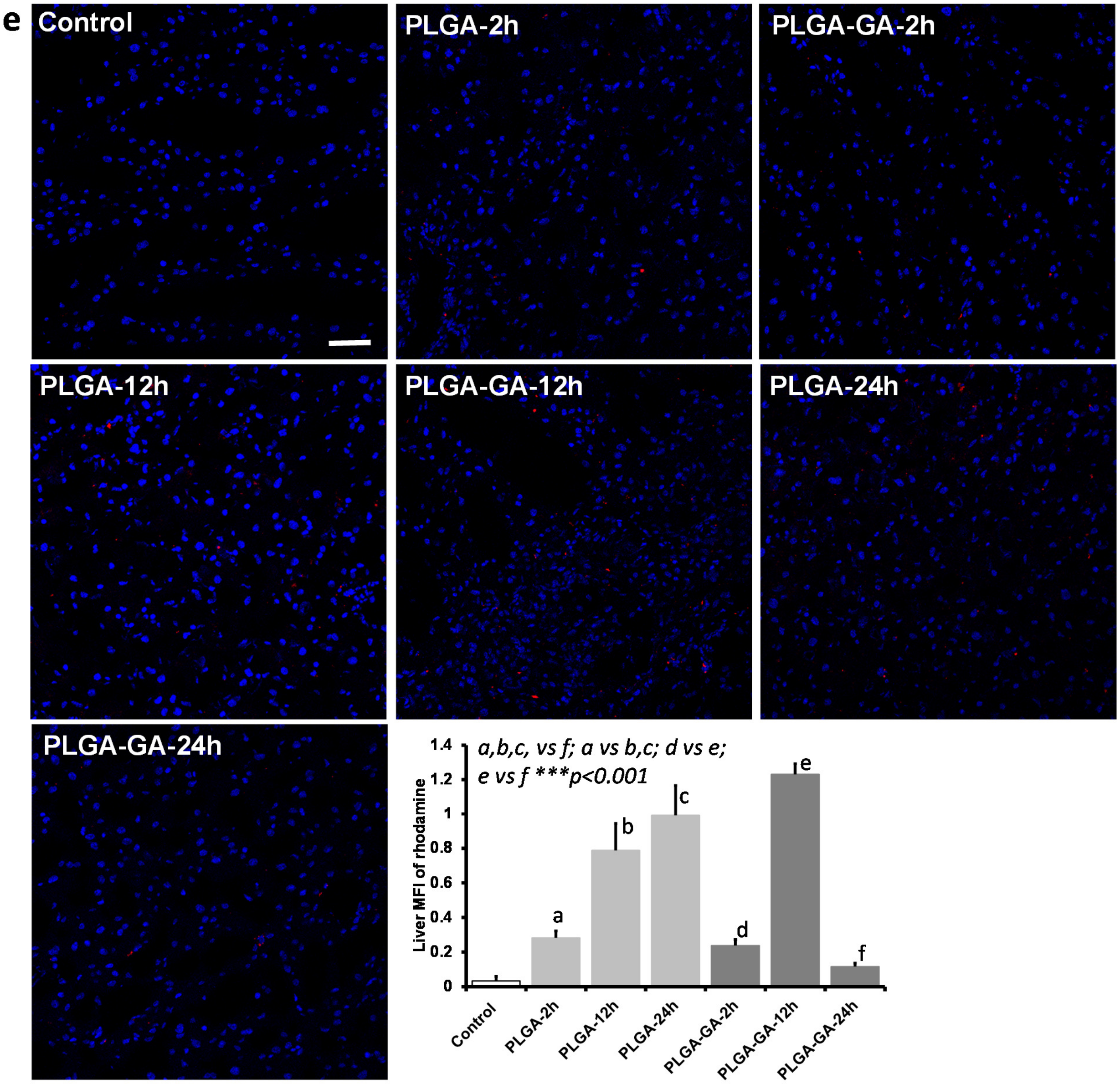
e
Control
PLGA-2h
PLGA-GA-2h
PLGA-12h
PLGA-GA-12h
PLGA-24h
PLGA-GA-24h
a,b,c, vs f; a vs b,c; d vs e;
e vs f ***p<0.001
Liver MFI of rhodamine
1.4
1.2
1
0.8
0.6
0.4
0.2
0
a
b
c
d
e
f
Control
PLGA-2h
PLGA-12h
PLGA-24h
PLGA-GA-2h
PLGA-GA-12h
PLGA-GA-24h

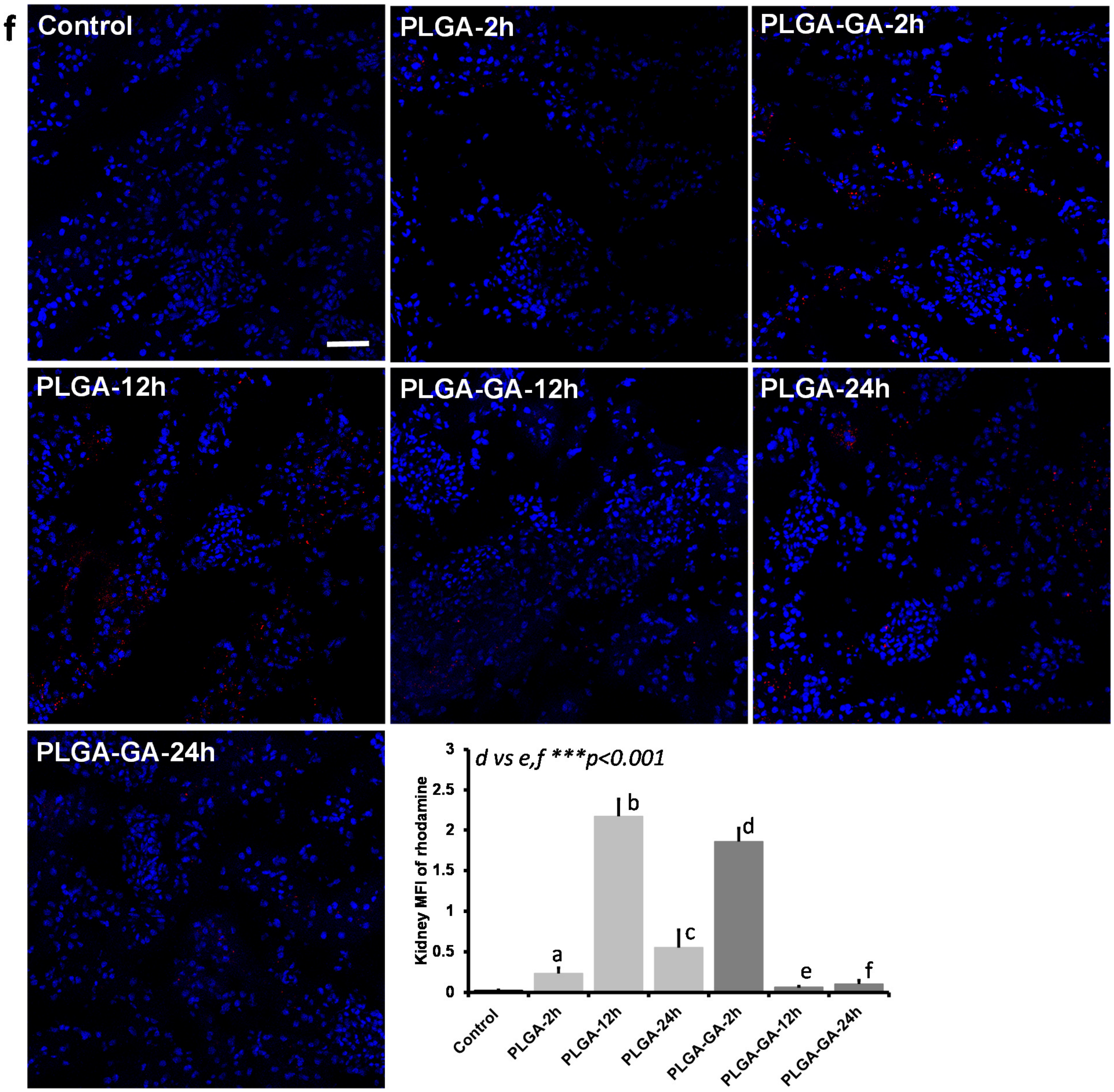
f
Control
PLGA-2h
PLGA-GA-2h
PLGA-12h
PLGA-GA-12h
PLGA-24h
PLGA-GA-24h
d vs e,f ***p<0.001
Kidney MFI of rhodamine
3
2.5
2
1.5
1
0.5
0
a
b
c
d
e
f
Control
PLGA-2h
PLGA-12h
PLGA-24h
PLGA-GA-2h
PLGA-GA-12h
PLGA-GA-24h

g

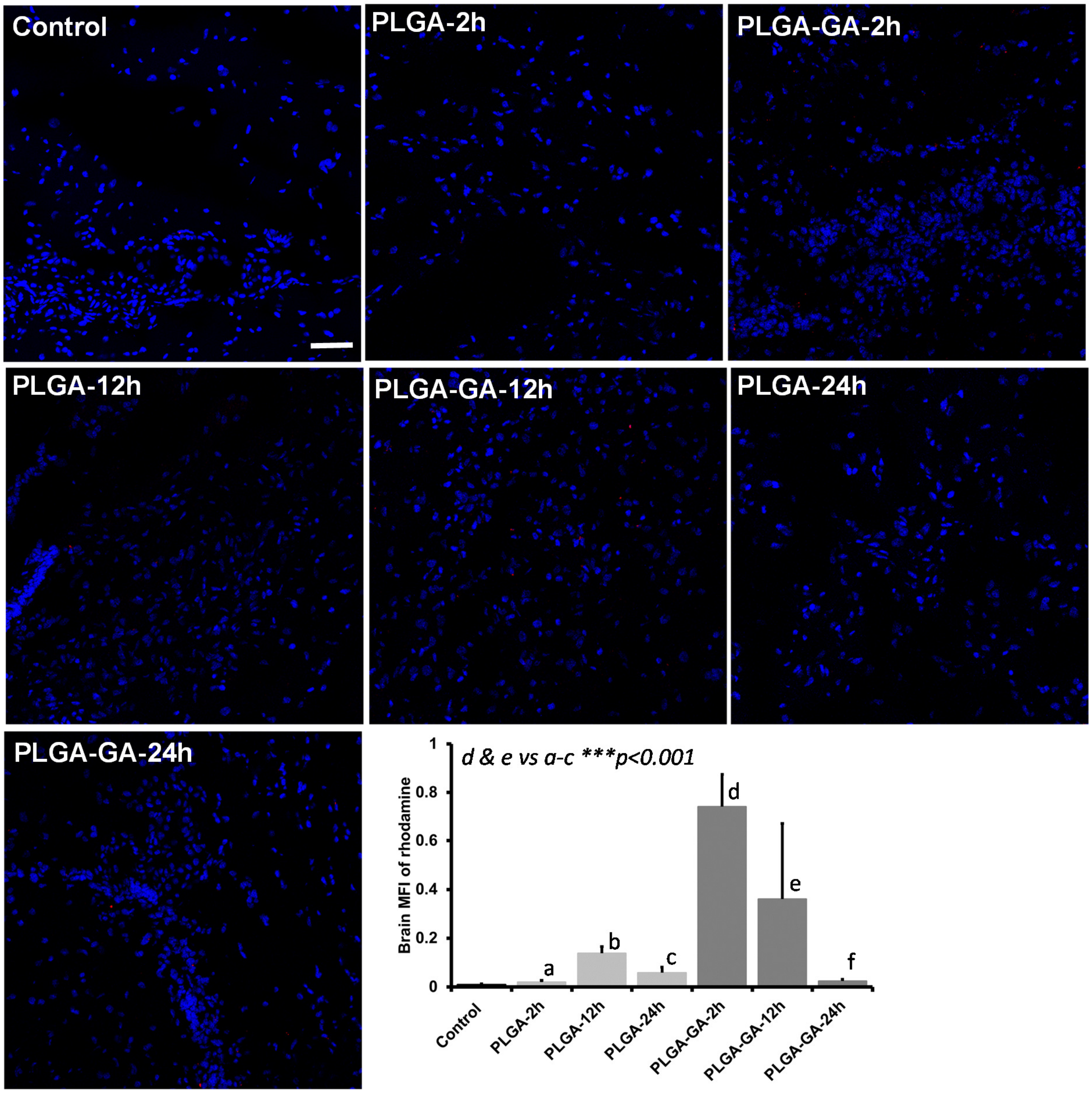

Figure S9. a-b, Excitation and emission spectra of plasma at 2, 12 and 24 h in SD rats after a single oral dose of PLGA100 and PLGA-GA100 NS. **c,** Image of the *in vivo* intestine sections presented in Figure 4d in main body of the paper with additional labeling of nuclei (blue) with DAPI. **d,** Spleen. **e,** Liver. **f,** Kidney, **g,** Brain, Nuclei are labeled blue (DAPI) in images d-g.

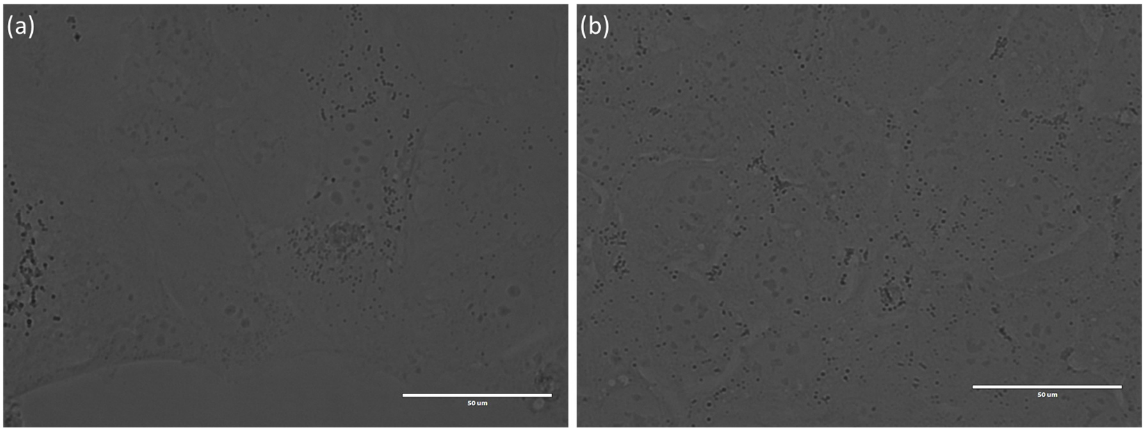

Figure S10. a, Normal protocol with media. **b,** protocol used in this study, 5 min incubation with 200 µl water followed by addition of media and continued 1 h incubation. The phase contrast images were taken using EOVS FL microscope at 60x, scale bar represents 50 µm and we did not find any changes in the cell morphology.

8. References

1 Lamprou, D. A., Venkatpurwar, V., and Kumar, M.N.V.R. Atomic force microscopy images label-free, drug encapsulated nanoparticles in vivo and detects difference in tissue mechanical properties in treated and untreated: A tip for nanotoxicology. *PLoS ONE,* **8,** e64490, doi:10.1371/journal.pone.0064490 (2013).
